# ToF-SIMS Depth Profiling of Metal, Metal Oxide, and Alloy Multilayers in Atmospheres of H_2_, C_2_H_2_, CO, and O_2_

**DOI:** 10.1021/jasms.1c00218

**Published:** 2021-12-22

**Authors:** Jernej Ekar, Peter Panjan, Sandra Drev, Janez Kovač

**Affiliations:** † Jožef Stefan Institute, Jamova cesta 39, SI-1000 Ljubljana, Slovenia; ‡ Jožef Stefan International Postgraduate School, Jamova cesta 39, SI-1000 Ljubljana, Slovenia; § Center for Electron Microscopy and Microanalysis, Jamova cesta 39, SI-1000 Ljubljana, Slovenia

**Keywords:** SIMS depth profiling, H_2_, C_2_H_2_, CO and O_2_ atmosphere, gas flooding, cluster secondary ions, matrix effect

## Abstract

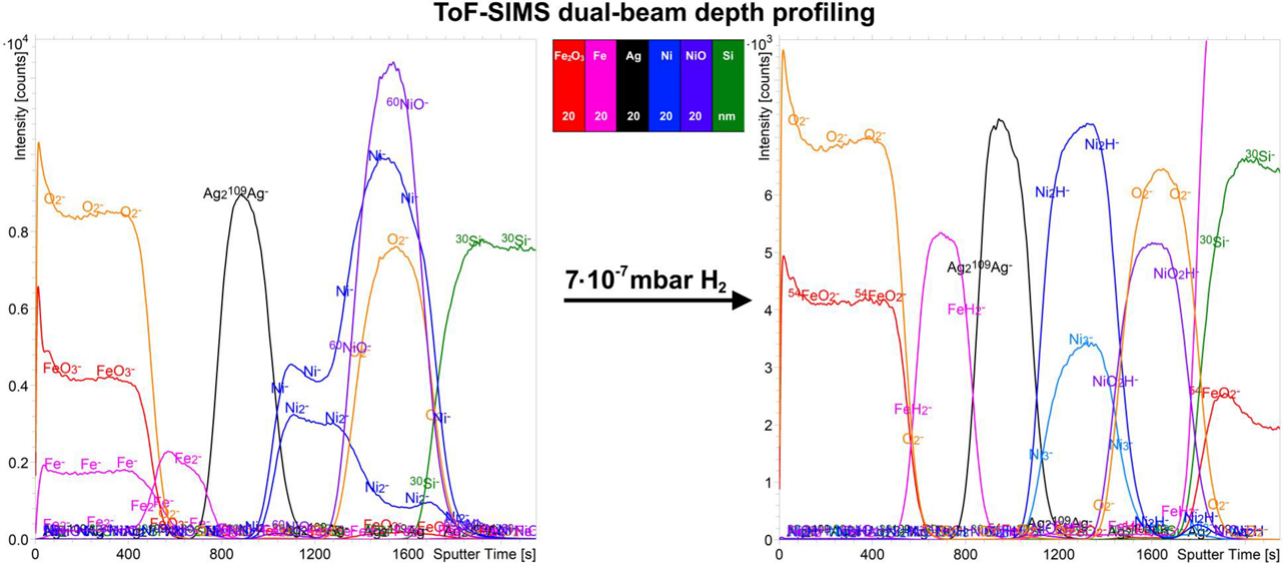

The influence of the flooding gas during ToF-SIMS depth profiling was studied to reduce the matrix effect and improve the quality of the depth profiles. The profiles were measured on three multilayered samples prepared by PVD. They were composed of metal, metal oxide, and alloy layers. Dual-beam depth profiling was performed with 1 keV Cs^+^ and 1 keV O_2_^+^ sputter beams and analyzed with a Bi^+^ primary beam. The novelty of this work was the application of H_2_, C_2_H_2_, CO, and O_2_ atmospheres during SIMS depth profiling. Negative cluster secondary ions, formed from sputtered metals/metal oxides and the flooding gases, were analyzed. A systematic comparison and evaluation of the ToF-SIMS depth profiles were performed regarding the matrix effect, ionization probability, chemical sensitivity, sputtering rate, and depth resolution. We found that depth profiling in the C_2_H_2_, CO, and O_2_ atmospheres has some advantages over UHV depth profiling, but it still lacks some of the information needed for an unambiguous determination of multilayered structures. The ToF-SIMS depth profiles were significantly improved during H_2_ flooding in terms of matrix-effect reduction. The structures of all the samples were clearly resolved while measuring the intensity of the M_*n*_H_*m*_^–^, M_*n*_O_*m*_^–^, M_*n*_O_*m*_H^–^, and M_*n*_^–^ cluster secondary ions. A further decrease in the matrix effect was obtained by normalization of the measured signals. The use of H_2_ is proposed for the depth profiling of metal/metal oxide multilayers and alloys.

## Introduction

Thin layers are often depth profiled when we want to analyze the chemical composition and thickness of a single layer or a multilayer structure. In this way, corrosion properties,^1−4^ diffusion mechanisms,^5^ native or artificially prepared oxide layers,^6,7^ layers that compose integrated circuits,^8−11^ and the composition of nanoparticles, nanolayers, and nanocomposite coatings,^12,13^ paints,^14^ adhesives,^15^ catalysts,^16,17^ thin polymer films,^18^ biological compounds^19,20^ and even cells and tissues^21,22^ can be studied. Such analyses can be performed using a variety of surface-sensitive analytical techniques such as SIMS (secondary ion mass spectrometry), XPS (X-ray photoelectron spectroscopy) or AES (Auger electron spectroscopy) in combination with ion sputtering for material removal.^23−25^ Laser ablation or plasma etching is also an option,^10^ but these approaches are less commonly applied. While depth profiling with ion sputtering is destructive, we can also use nondestructive methods such as RBS spectroscopy,^26^ ellipsometry, X-ray reflectometry,^27^ or angle-resolved analyses such as AR-XPS. When thicker layers are of interest, sectioning of the material either via mechanical means, such as a microtome^28^ or with a focused-ion beam (FIB),^29^ is preferred. Cross sections prepared in this way can be analyzed using, for example, a scanning electron microscope (SEM)^29^ or SIMS imaging.^28^

With ion-sputtering methods, there are many possible ions to choose between. Ar^+^ ions are the most frequently used.^25,30−32^ Other options include Xe^+^ ions^20,33,34^ and the reactive Cs^+^ and O_2_^+^ ions.^30,31^ Cs^+^ ions increase the yield of negative secondary ions in SIMS spectrometry due to their incorporation into the surface of the sample with a consequent reduction of the work function.^31^ Cs^+^ ions also make possible the analysis of cluster secondary ions, which are mainly formed as MCs_*n*_^+^,^35,36^ where M stands for any metal atoms. On the other hand, O_2_^+^ ions increase the yield of positive secondary ions during SIMS depth profiling.^37^ If appropriate ion sources are used, O^–^ primary ions can also be used for sputtering, but this method is not often practiced.^31^

As these mono- or diatomic primary ions penetrate deep into the sample, causing extensive damage to the chemical bonds and the mixing of atomic/molecular layers, larger molecular as well as cluster primary ions were introduced, especially for molecular depth profiling.^19,38−41^ The energy of the monatomic primary ions is focused on a small radius crater and deep in the subsurface layers, damaging and breaking molecules yet to be analyzed.^19,25,39^ The energy of the polyatomic and cluster primary ions is distributed among all its constituent atoms and deposited mainly on the surface, resulting in less damage.^39−41^ Consequently, the most commonly applied energies for monatomic primary ions are in the range of a few 100 eV up to a few keV,^11,37,42^ while for polyatomic ions they are in the range of a few keV up to a few 10 keV, mainly depending on their type.^19,22,25,40^ The first widely applied polyatomic source was SF_5_^+^, closely followed by the fullerenes, C_60_^+^.^19,22,40^ More recently, Ar cluster ions have gained popularity due to their versatility, both in terms of energy (a few keV to a few 10 keV) and size, as they can be made up of 100–5000 Ar atoms.^25,40,43^ Also worth mentioning are the experiments with H_2_O,^40^ CO_2_,^43^ and O_2_ clusters.^44^

Since we are focusing on SIMS depth profiling, we should first distinguish between single-beam and dual-beam depth profiling. In single-beam depth profiling, the same ion beam is used for the etching process and analysis, while in dual-beam depth profiling, different ion beams are used for the analysis and ion etching.^41,45^ The analysis is most commonly performed with a liquid metal ion gun (LMIG) using Bi^+^, Bi_3_^+^, Au^+^, Au_3_^+^, Ga^+^, or In^+^ primary ions.^31^ Dual-beam depth profiling offers an advantage because the ion beam is optimized for the analysis. On the other hand, all of the material sputtered during the etching process is lost.^41^ In addition to the deterioration of the depth resolution and ion-bombardment-induced damage, the application of SIMS is also limited by the matrix effect and the nonconstant ionization yields.^46−49^ The matrix effect is the effect of the substrate on the ionization probability of the particles emitted from the surface, both increasing or decreasing the ionization yield of either positive or negative secondary ions depending on the substrate from which they originate.^46−50^ The matrix effect is particularly critical for (semi)quantitative analyses.^48,50,51^

Fortunately, there are many ways of reducing the matrix effect. One commonly applied approach is laser postionization of the neutral sputtered particles (laser-SNMS).^52−54^ Since the particles are ionized in the plume, there is no matrix around them to affect the ionization.^53^ Laser-SNMS can be used in depth profiling,^55^ thereby improving the ionization yield.^56,57^ With this method, we can selectively ionize only some of the neutral particles (resonant laser-SNMS)^58^ or nonselectively ionize all of them (nonresonant).^52^ In the case of resonant laser-SNMS, we must know exactly what is being analyzed, and in the case of the nonresonant approach, we must make sure that the laser intensity is high enough to ionize all the particles.^59^ The sputtered neutrals can also be postionized with an electron beam, similar to a laser.^60−63^ We can also intentionally exploit the matrix effect by depositing a thin metal layer, usually Au or Ag.^64,65^ However, depth profiling combined with metal-assisted SIMS can only be performed while using a nonconventional experimental setup.^66^ The ionization yield can also be increased by depositing ionic liquids^67^ or graphene oxide^68^ on the sample surface. Furthermore, the samples can be mixed with specific matrices,^69−71^ but with this approach we lose the surface sensitivity. Results similar to those using metal-assisted SIMS, but also applicable for depth profiling, offer sputtering with Cs^+^ or cosputtering with Cs^+^ and Xe^+^.^36,72^ The metal that increases the ionization yield in this case is cesium, which opens the possibility of analyzing the MCs_*n*_^+^ secondary-ion clusters.^36,72^ More recently, dynamic reactive ionization (DRI) has been developed in which reactive HCl molecules are incorporated into the Ar cluster primary ions.^73−75^ Water on the sample surface introduced into the analysis chamber through the gas valve enhances the protonation and consequently increases the ionization yield while reducing the matrix effect.^73,74^ DRI is compatible with depth profiling.^75^ Finally, it is also possible to flood different gases into the analysis chamber during both surface analysis and depth profiling, thus introducing a gaseous matrix that is universal, regardless of the sample used.^76−79^ The most commonly used gas is O_2_, leading to higher ionization yields for positive secondary ions^76,77^ or improving the depth resolution while reducing surface roughening.^78^ The ionization yield can also be affected by the introduction of some other compounds, such as water or fluorine (via XeF_2_), which also change the sputtering rate.^79^

The aim of our research was to investigate the influence of different gases on the quality of the SIMS depth profiles of different multilayer structures. Our study was performed on a ToF-SIMS instrument with a dual-beam configuration. Using Bi^+^ ions for the analysis and Cs^+^, O_2_^+^, or Ar^+^ for the etching process, we aimed to optimize the depth profiles recorded while changing the atmosphere in the analysis chamber. For our study, we selected three different multilayer structures similar to those we frequently analyze in our laboratory for industrial and academic partners. Our goal was to find a way to clearly and unambiguously resolve the layered structure of the samples mainly by manipulating the atmosphere in the analysis chamber. Using this process, the ionization yield of the secondary ions and the matrix effect would be turned in our favor. In our study, we compared the effects of O_2_, CO, C_2_H_2_, and H_2_. The results of our experiments show that the introduction of H_2_ gas during the SIMS depth profiling improves the chemical sensitivity of the SIMS method, provides a clear distinction between the metallic and metal-oxide layers, allowing the easier identification of elements and their oxides in thin films, and improves the depth resolution.

## Experimental Section

### Preparation of Multilayered Samples

All of the metals and metal oxides were deposited in a Sputron triode sputtering system (Balzers Oerlikon). The background pressure was better than 1 × 10^–6^ mbar. The partial pressure of the argon working gas in the vacuum chamber was 2 × 10^–3^ mbar for all the processes. A maximum substrate temperature of less than 100 °C was maintained during the deposition. A quartz-crystal microbalance was used to calibrate the deposition rates. The deposition rates and thickness reproducibility were better than 2%.

The 60 mm diameter targets are interchangeable *in situ*, allowing us to easily prepare multilayer structures without interrupting the vacuum from one deposition to the next. All the targets were initially cleaned for 5 min to remove the native oxide and other impurities on their surfaces. High-purity targets were used as the sputtering source. With an average DC power of 60 W/cm^2^ (1700 V/0.6 A) on the pure metal target (Al, Fe, Ti, Cr, Ni), deposition rates in the range 10–12.5 nm/min were achieved. At the same target power, the deposition rate of the TiSi (50/50) was slightly lower (7.6 nm/min). At half the target power, the deposition rate of Ag was much higher (around 16 nm/min). Silicon was RF sputtered onto the target at 36.5 W/cm^2^ ,and a deposition rate of 3.1 nm/min was achieved. For depositing the layers of Ti–Si alloys, composite targets were used with 3:1 and 1:1 atomic ratios between the Ti and Si.

Metal oxide layers (Cr_2_O_3_, TiO_2_, Al_2_O_3_, Fe_2_O_3_, NiO) were prepared by reactive sputtering. In this process, thin oxide films were deposited on substrates by sputtering metallic targets in the presence of oxygen mixed with an argon working gas. The composition of the deposited layers and the deposition rate are very sensitive to the supply of oxygen; therefore, the flow rate of the oxygen (99.998%) was controlled with a flowmeter. Prior to the deposition, the targets were cleaned and conditioned in pure Ar plasma and Ar/O_2_ plasma at a closed shutter, respectively. Due to the lower density of the oxide layers, the deposition rates were higher than for pure metal layers at the same power on the target (from 11.4 nm/min for Al_2_O_3_ to 26.5 nm/min for NiO).

### SIMS Measurements

The ToF-SIMS analyses were made using a TOF.SIMS 5 instrument from ION TOF. The instrument has two ion guns (dual-beam depth profiling), a ToF analyzer with a reflectron, a microchannel plate (MCP) detector and a low-energy electron gun. We used Bi^+^ primary ions from a BiMn LMIG field-ionization source as the analysis beam with an energy of 30 keV and a current between 0.9 and 1.2 pA, depending on the measurement. The Bi^+^ primary ion beam was pulsed with a pulse length of 5.9 ns. With the settings used, the analyzed depth was about 2 nm, the detection limits around 1 ppm, and the mass resolution *m*/Δ*m* between 8000 and 20000, depending on the peak of interest. Only the mass resolution of the H^–^ signal was lower, around 3000. For the depth profiling we used a Cs^+^ or O_2_^+^ sputter ion beam with an energy of 1 keV for both ion species. In the case of the Cs^+^ sputter beam, the ion current ranged from 42 to 58 nA, while in the case of the O_2_^+^ it ranged from 107 to 116 nA, depending on the measurement. The surface-ionization-based ion source and the electron-impact ion source were used for the generation of the Cs^+^ and O_2_^+^ ions, respectively. The analyses with the Bi^+^ primary ions were performed over a 50 × 50 μm scanning area (128 × 128 pixels), located in the center of the 400 × 400 μm etching crater created by the Cs^+^ or O_2_^+^ sputter ion beam. Secondary ions were analyzed over the *m*/*z* range from 0 to 875.

The gases used during the depth profiling were introduced into the analysis chamber close to the analyzed region (a distance of less than 1 cm). The introduction of the gas was manually controlled with a precise gas-leak valve that had a Swagelok installation and a capillary leading toward the analyzed area. We used gases with different purity levels, i.e., 6.0 H_2_, industrial grade C_2_H_2_, 3.0 CO, and 5.0 O_2_. The applied pressures were 7 × 10^–7^ mbar for H_2_, 2 × 10^–7^ mbar for C_2_H_2_, 2 × 10^–7^ mbar for CO, and 8 × 10^–8^ mbar for O_2_. These were approximately the lowest pressures needed to saturate the formation of cluster secondary ions from the metals and the respective gas. Since optimizing the gas pressures was not the main goal of our study, we emphasize that it is probably possible to further reduce the pressures of the gases.

### Cross-Section Sample Preparation and TEM Measurements

The sample was cut into blocks and mounted face to face in a brass ring with epoxy glue. The TEM specimen was ground to a thickness of 100 μm and dimpled down to 15 μm at the disc center (Dimple grinder, Gatan, Inc., Warrendale, PA). The TEM specimen was finally ion-milled (PIPS, Precision Ion Polishing System, Gatan, Inc., Warrendale, PA) using 3 kV Ar^+^ ions at an incidence angle of 8° until perforation. Detailed structural investigations of the sample were performed using a 200 kV transmission electron microscope with field emission electron gun (JEM-2010F, Jeol Ltd., Tokyo, Japan).

## Results and Discussion

### Samples

**Figure 1:**
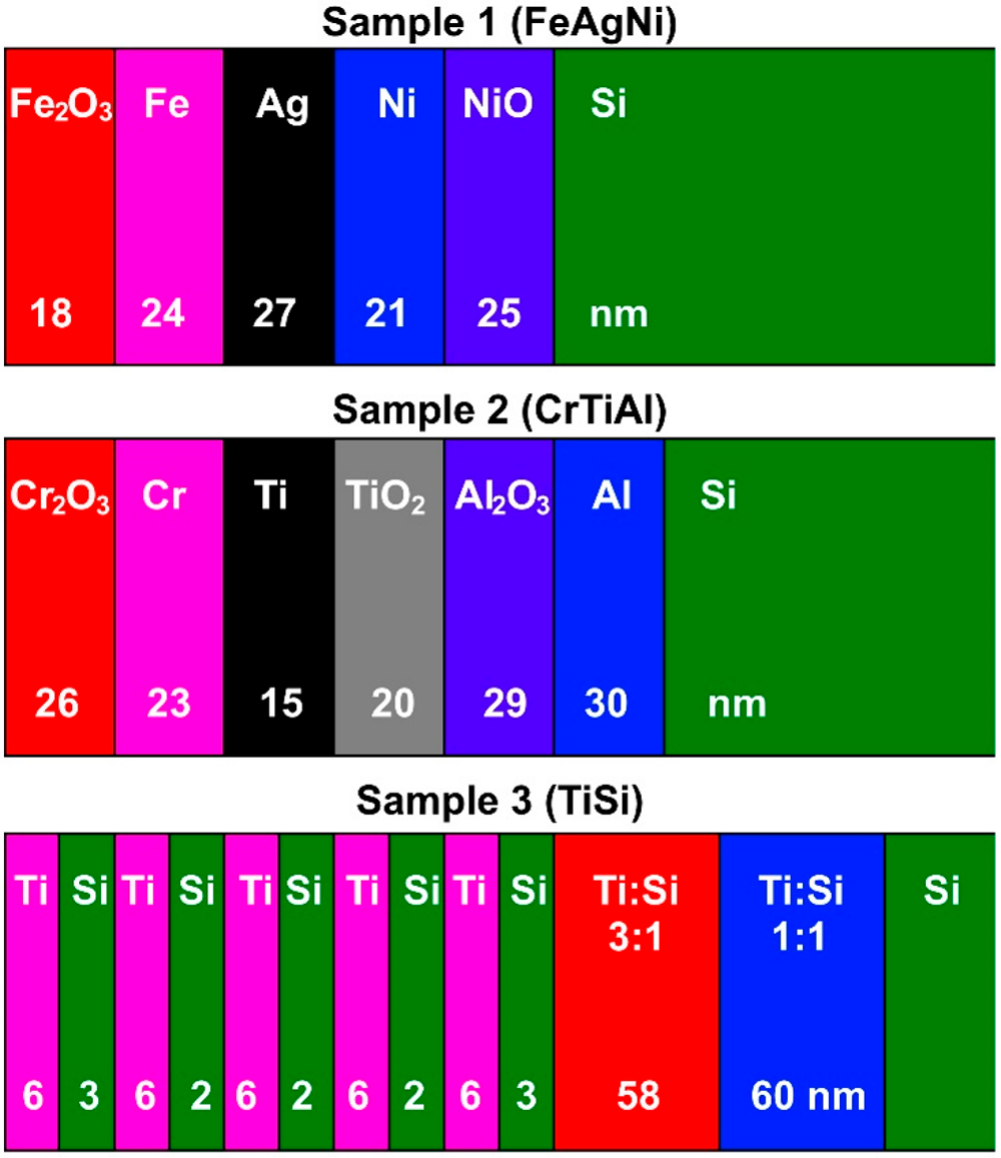
Schematic presentation of sample 1 (FeAgNi), sample 2 (CrTiAl), and sample 3 (TiSi).

We conducted our study on the influence of different gases during SIMS depth profiling on three different samples. Samples 1 (FeAgNi) and 2 (CrTiAl) were multilayer structures of Fe_2_O_3_/Fe/Ag/Ni/NiO and Cr_2_O_3_/Cr/Ti/TiO_2_/Al_2_O_3_/Al, respectively. Both were prepared on mirror-like polished silicon wafers. All the layers had a thickness between 15 and 30 nm, with the exact values given in Figure 1. The thicknesses of the layers were determined by TEM imaging, with the corresponding images presented in Figure S2. The third sample (TiSi), also shown in Figure 1, consisted of 10 alternating layers of Ti and Si, only 5.5 and 2.5 nm thick, respectively. They were followed by two layers of Ti–Si alloy with a thickness of almost 60 nm. In the first TiSi layer, the atomic ratio between Ti and Si was 3:1 with the higher concentration being titanium, while in the second TiSi layer this ratio was 1:1. A multilayer structure was again deposited on the silicon wafer.

In the first part of our study, we measured the SIMS depth profiles of the three samples described using a dual-beam depth profiling technique without the presence of an additional gas. In the second part, we introduced gases such as O_2_, CO, C_2_H_2_, and H_2_ and qualitatively compared the acquired depth profiles.

### O_2_^*+*^ Sputtering

**Figure 2:**
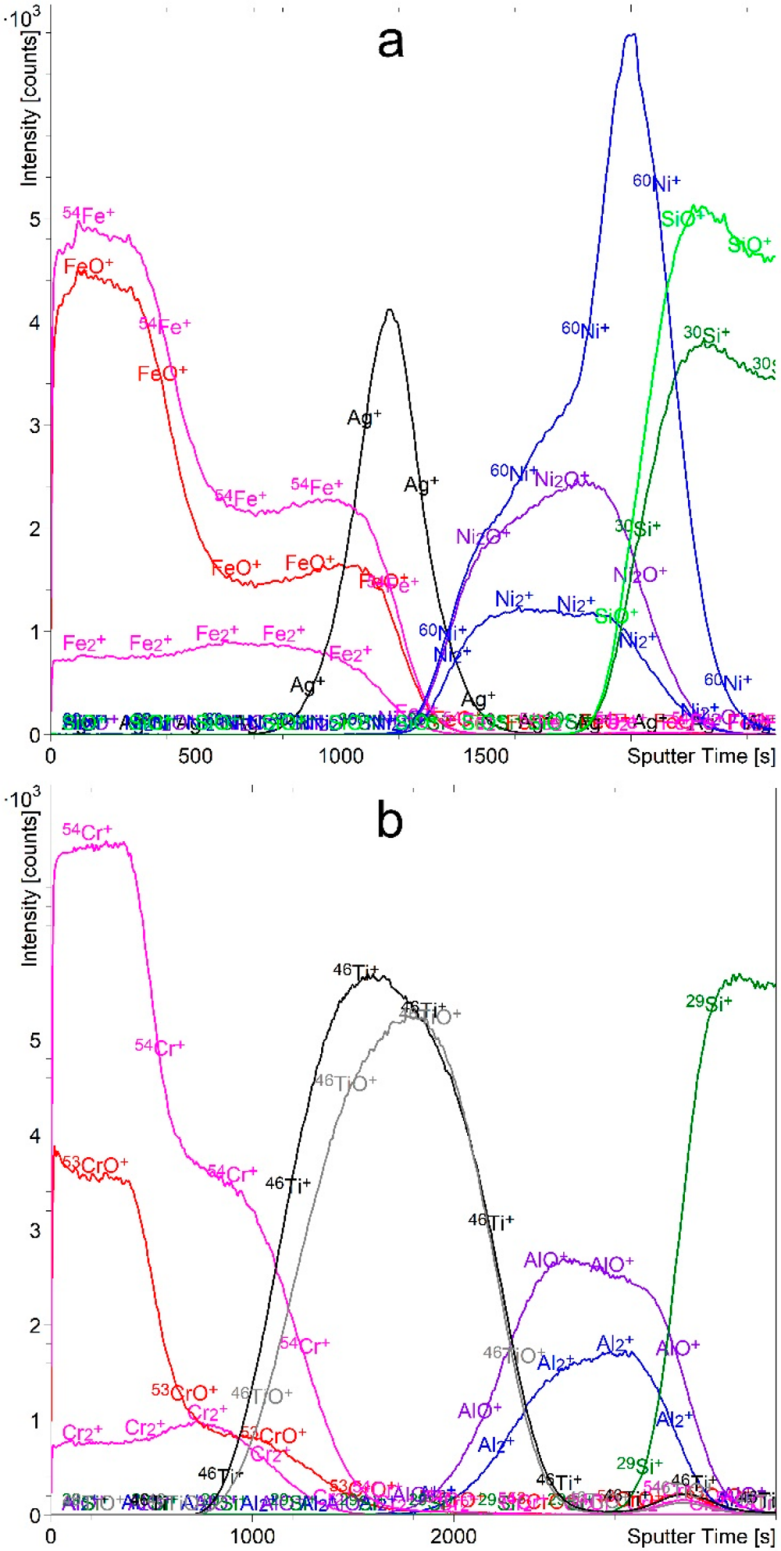
Depth profiles of FeAgNi (a) and CrTiAl (b) samples recorded using a 1-keV O_2_^+^ sputtering beam. Intensities of some signals were multiplied by a specific factor as a way of reducing the intensity scale interval (0.6 for ^54^Fe^+^, 0.5 for ^60^Ni^+^, 0.7 for SiO^+^, 0.7 for ^54^Cr^+^, 0.3 for ^46^Ti^+^, and 0.3 for ^46^TiO^+^).

For the initial depth analyses we used an O_2_^+^ beam for etching. Figure 2 shows the depth profiles of the positive secondary ions of the FeAgNi and CrTiAl samples using a 1 keV O_2_^+^ sputter beam. As is evident from Figure 2, the difference in the intensity of the metal oxide secondary-ion signals (MO^+^) between the layers of pure metal and metal oxide of the same element can only be seen for Fe and Cr. For the other elements (Ni, Ti and Al), the intensity of their MO^+^ signal is relatively constant through both the metal and its oxide layers due to the oxidation caused by the O_2_^+^ ions. Additionally, due to the matrix effect, the M^+^ signals either show a higher intensity in the layer of their oxide rather than in the layer of the pure metal (Fe, Ni, and Cr) or have a constant intensity through both layers (Ti) (Figure 2). However, this is not the case for the Al^+^ signal, which was saturated in our measurements. The M_2_^+^ signals are much less affected by the matrix and therefore give a somewhat more representative picture, but they still do not provide an adequate description of the composition of the layers. For some metals, such as Ti, the Ti_2_^+^ signal is also too weak to be considered relevant. From the depth profiles recorded when using O_2_^+^ ions, only different metal layers can be distinguished from each other, but not the layers of the pure metal and its oxide.

The use of O_2_^+^ ions for etching was partially successful in the analysis of the TiSi sample, where we can recognize the multilayer structure of this sample (Figure S2a). Both the M^+^ and MO^+^ signals are correctly positioned and exhibit relatively intense maxima and minima. But we encounter a problem when we compare Ti–Si alloy layers with different Ti/Si concentration ratios. As the relative concentration of Si increases, we also observe an increase in the intensity of the Si^+^ and SiO^+^ signals, as shown in Figure S2b. On the other hand, as the relative concentration of Ti decreases, there is no clear decrease in the intensity of the Ti^+^ signal, while the intensity of the TiO^+^ signal decreases only slightly. Therefore, the application of O_2_^+^ ions for sputtering is not suitable when analyzing multilayer structures composed of metal layers and their oxides as well as when analyzing multilayer structures composed of alloy layers with different compositions.

### Cs^+^ Sputtering

**Figure 3:**
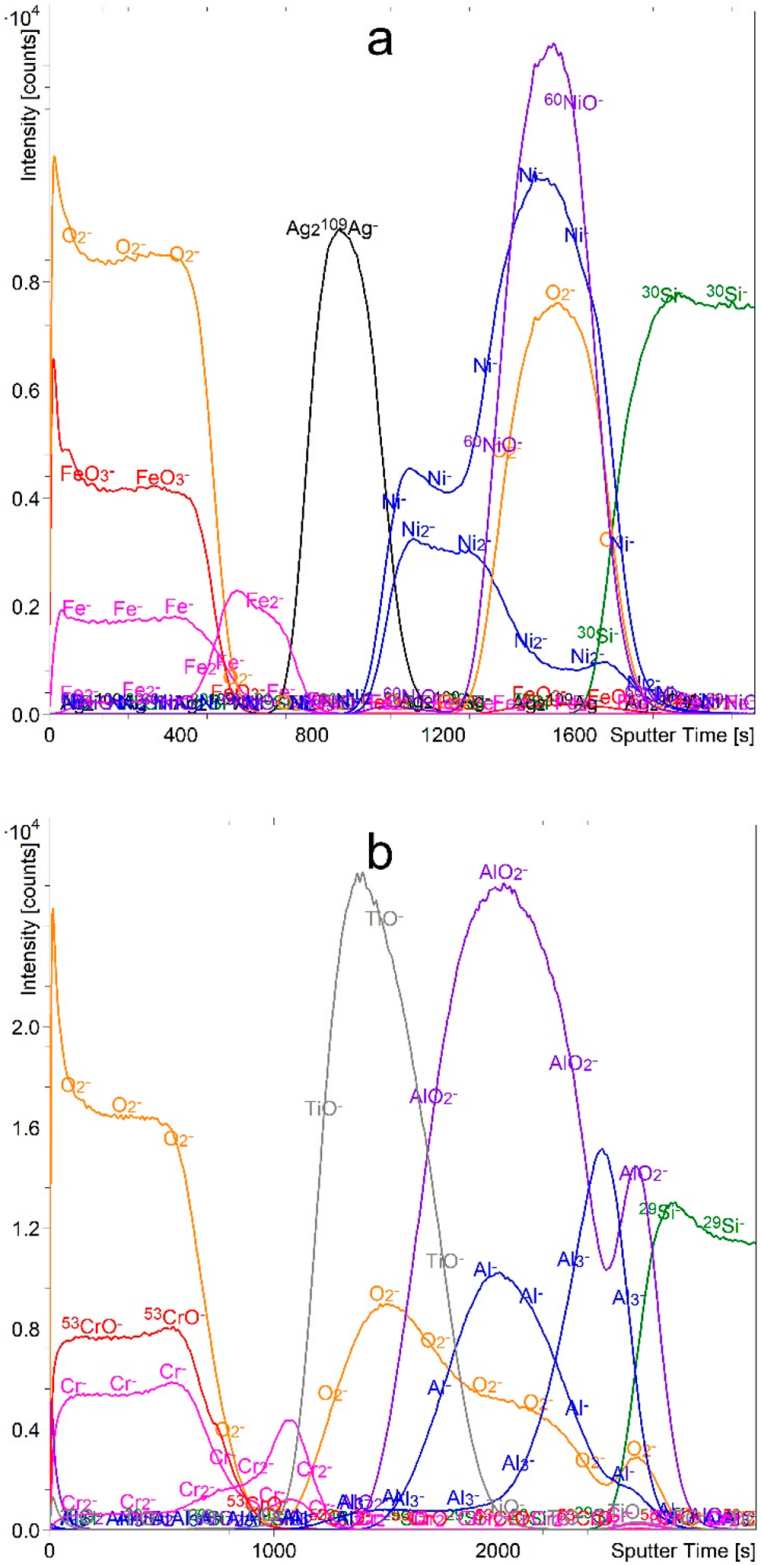
Depth profiles of FeAgNi (a) and CrTiAl (b) samples recorded using a 1 keV Cs^+^ sputtering beam. The intensity-multiplication factors are 0.3 for O_2_^–^, 2.0 for Fe_2_^–^, 0.4 for Ni^–^, 0.3 for ^60^NiO^–^, 4.0 for Cr_2_^–^, and 0.5 for TiO^–^.

In the next experiments, the 1 keV Cs^+^ ion beam was used for etching. Figure 3 shows the depth profiles of the negative secondary ions sputtered from the FeAgNi and CrTiAl samples. From the depth profiles measured using Cs^+^ ions, oxide layers can be identified from both the O_2_^–^ and MO_n_^–^ signals. However, there are still problems with the identification of metal layers. Indeed, the analysis of the depth profiles in Figure 3 shows that all of the M^–^ signals have maxima in the respective oxide layer, except for Ti. The intensities of both the Ti^–^ and Ti_*n*_^–^ signals are too low to treat them as relevant for the analysis. The M_*n*_^–^ secondary ions are much less affected by the matrix effect than the M^–^ ions and show maxima in the layers of the pure metals (Figure 3). This observation implies that the matrix has a greater effect on the monatomic secondary ions than on the cluster secondary ions. However, the intensity of the M_*n*_^–^ ions is still significant in the oxide layers. Moreover, with the exception of Al_*n*_^–^, their average intensity is very low so that the signal-to-noise ratio is also low.

**Figure 4:**
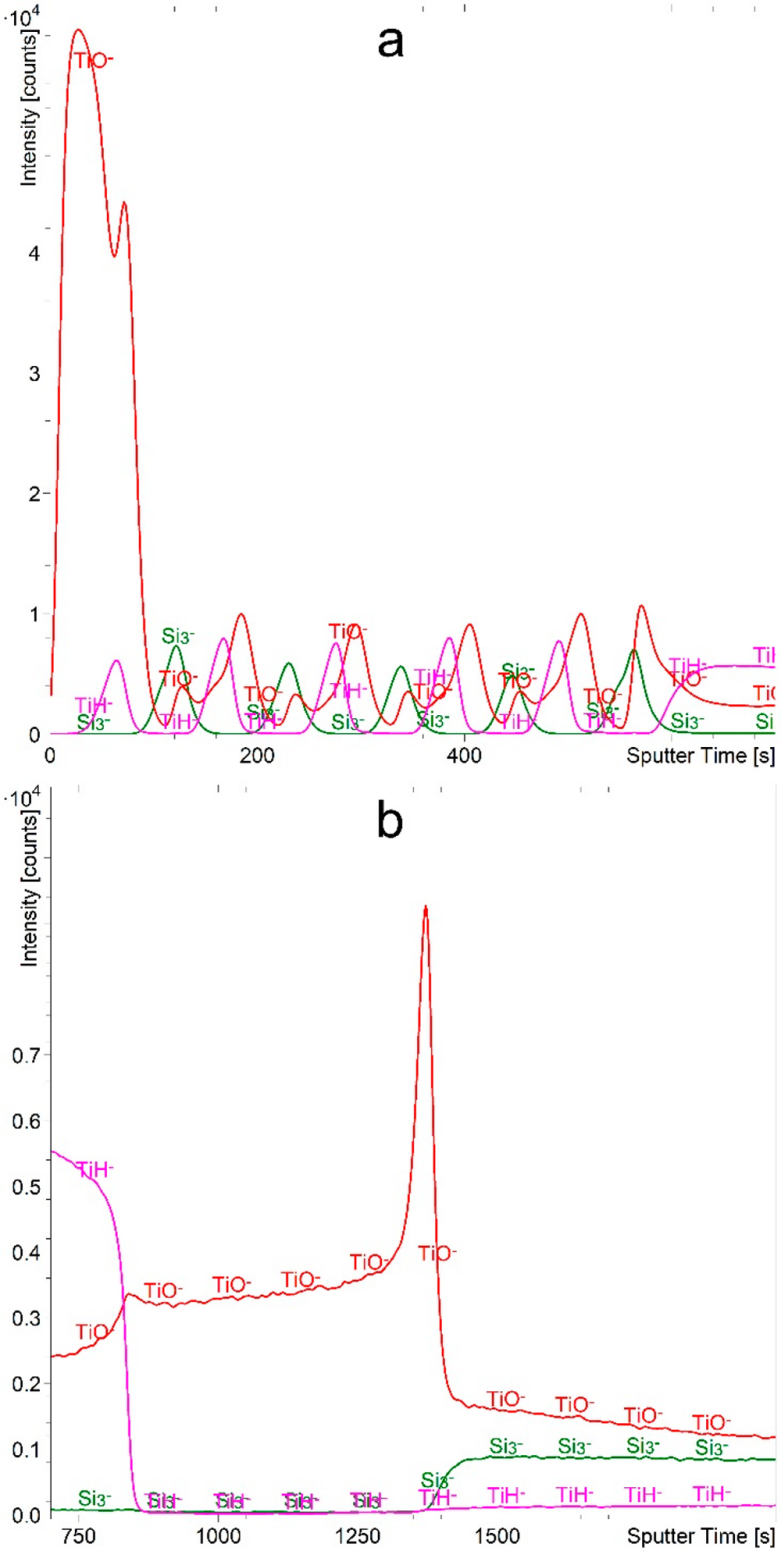
Depth profile of TiSi sample recorded using a 1 keV Cs^+^ sputtering beam. The depth profile (a) presents the first 700 s of etching time, while the profile (b) presents the etching time interval between 700 and 2000 s.

The application of the Cs^+^ beam for sputtering the TiSi sample also showed some problems regarding the interpretation of the measured depth profiles. The Si layers in the multilayer structure can be easily identified via the Si_3_^–^ signal (Figure 4a). The TiO^–^ signal, on the other hand, has a disproportionately intense maximum in the first Ti layer and an additional maximum in the last Si layer. But the TiH^–^ signal resolves the multilayer structure more clearly. This presents the only case of an intense hydride signal formation, even without the presence of H_2_ during the analysis. Namely, other metals and even Si in the TiSi sample form different and much more intense hydride signals when H_2_ gas flooding is applied. Furthermore, even the Ti in the CrTiAl sample does not form hydride signals without H_2_ being present in the analysis chamber. We can therefore conclude that the thin Ti and Si layers are permeable to the hydrogen, while the Cr and Cr_2_O_3_ layers protect the underlying Ti from the hydrogen. Since we observed TiH^–^ formation for two different sample series while they were exposed only to the ambient conditions, we can conclude that measurable amounts of hydrogen dissolve in the thin Ti layers even when the only source of hydrogen is the air. This phenomenon can be explained by the good solubility of hydrogen in titanium.^80,81^ The ambient source of the hydrogen can even be water, as studies have shown that water forms a metal oxide layer on the surface while hydrogen atoms diffuse deeper into the metal, forming hydrides.^82,83^ It should be also mentioned that hydrogen, either from the H_2_O or H_2_ molecule, dissociates into atoms before it diffuses into the metal.^84,85^

Furthermore, the layers of the Ti–Si alloys show unsatisfactory results as well (Figure 4b). It appears that the hydrogen is dissolved at the beginning of the first alloy layer as well, but the abrupt fall in the intensity of the TiH^–^ signal at around 840 s of sputtering (Figure 4b) indicates that the hydrogen cannot penetrate deeper. Furthermore, with a decreasing relative concentration of Ti, the intensity of the TiO^–^ signal also decreases, but the prominent TiO^–^ maximum at the interface between the alloys severely alters the depth profile. Only the Si_3_^–^ (and Si_2_^–^) signals correctly describe both alloys. Namely, with an increasing relative concentration of Si, the intensity of both the Si_3_^–^ and Si_2_^–^ signals also increases. Some anomalies and artifacts seen in Figure 4 can also be explained with the change in the concentration of implanted Cs atoms when crossing interfaces as well as with the oxidized species at the interfaces originating from the sample preparation. Both of these phenomena strongly influence the ionization probability.

**Figure 5:**
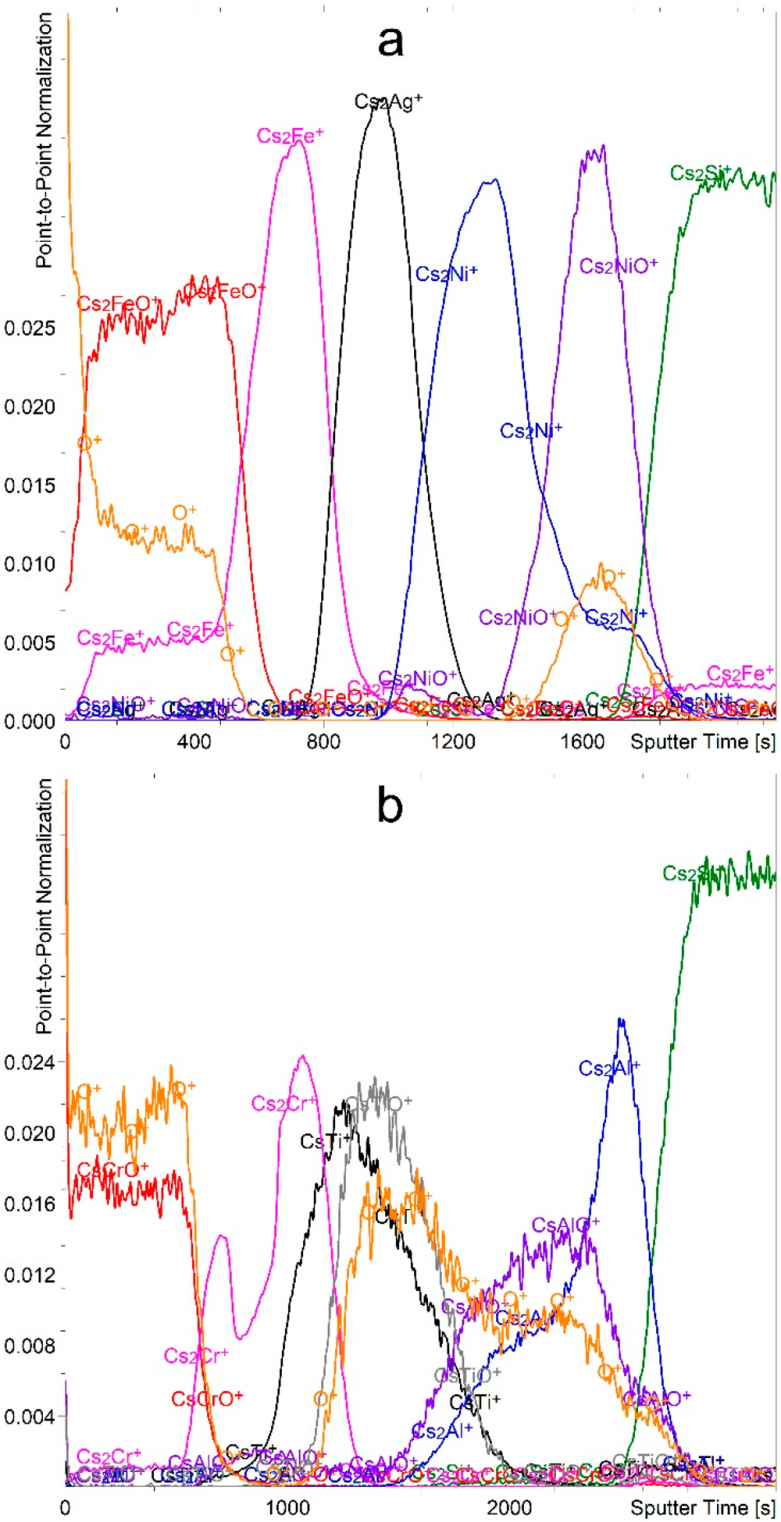
Depth profiles of FeAgNi (a) and CrTiAl (b) samples with the scale normalized to the intensity of the Cs_2_^+^ signal recorded using a 1-keV Cs^+^ sputtering beam. The intensity-multiplication factors are 3.0 for O^+^, 2.0 for Cs_2_FeO^+^, 0.15 for Cs_2_Ag^+^, 0.3 for Cs_2_Ni^+^, 3.0 for Cs_2_NiO^+^, 0.2 for Cs_2_Cr^+^, 2.0 for CsTiO^+^, 2.0 for CsAlO^+^, and 0.4 for Cs_2_Al^+^.

If Cs^+^ ions were used for the etching, the positive secondary ions have a very low intensity and are strongly influenced by the matrix effect originating from the Cs implantation and the sample oxidation during preparation. On the other hand, Cs_2_M^+^ and Cs_2_MO^+^ cluster ions show better results and are often used in the so-called MCs^+^ approach.^35,36^ These signals can be normalized to the Cs^+^ or Cs_2_^+^ signals. In our case, normalization to the Cs^+^ signal is not effective because the Cs^+^ signal is saturated. The correct multilayer structure of the FeAgNi sample can be determined from the depth profiles based on the Cs_2_M^+^ and Cs_2_MO^+^ secondary ions (Figure 5a). The O^+^ ions provide additional confirmation. The CrTiAl sample, on the other hand, appears to be much more problematic (Figure 5b). Namely, the Cs_2_Cr^+^ signal shows two maxima, the CsTi^+^ and CsTiO^+^ signals do not offer a clear distinction between the pure Ti and TiO_2_, and the much-less-intense Cs_2_Ti^+^ and Cs_2_TiO^+^ signals do not offer much better results. Finally, neither the CsAlO^+^ nor Cs_2_AlO^+^ signals provide a clear insight into the Al_2_O_3_/Al layers. There are also issues that occur with both the FeAgNi and CrTiAl samples. Namely, the CsM^+^ signals are not representative because they are strongly affected by the matrix effect and thus show maxima in the oxide layer. Moreover, the Cs_2_M^+^ signals also do not disappear in the oxide layers. Last but not least, most of the Cs^+^ cluster signals are of very low intensity. They are one to two orders of magnitude weaker than the signals of the positive secondary ions recorded while etching with O_2_^+^ ions or the negative secondary ions recorded while etching with Cs^+^.

Significant problems were encountered in the analysis of the TiSi sample, too. The Cs_2_Ti^+^ and Cs_2_Si^+^ signals show only partial and unclear differentiation of the Ti and Si thin layers, while the CsTi^+^ shows no clear maxima or rather too many of them (Figure S3a). The CsSi^+^ signal shows the same pattern as the Cs_2_Si^+^ signal, only it is less intense. Due to the very low intensity of the Cs_2_Ti^+^ signal in particular, we are faced with many artifacts as well as with secondary misplaced maxima. The discrimination between the Ti–Si alloy layers with different Ti/Si atomic ratios is slightly better, as the intensity of the Cs_2_Ti^+^ signal decreases when the relative concentration of the Ti decreases and the intensity of the Cs_2_Si^+^ signal increases when the relative concentration of the Si increases (Figure S3b). This is not true for the CsTi^+^ and CsSi^+^ signals, as their intensity remains more or less constant. Additionally, we again observe an increase in the intensity of both the Cs_2_Ti^+^ and Cs_2_Si^+^ signals for an etching time of about 1100 s caused by the abrupt fall in the concentration of the dissolved hydrogen. Finally, the consequence of the very low intensity of all these signals is a significant presence of noise in the lines of the depth profile.

### Gas Flooding: General and O_2_

From the results shown above, we can conclude that upon sputtering with only O_2_^+^ or Cs^+^ ions, we cannot unambiguously determine the layered structure of multilayer samples composed of metals and their oxides nor can we find the differences between alloys of the same constituents with their different relative concentrations. Even a combination of the depth profiles of positive secondary ions (recorded during sputtering with O_2_^+^ or as MCs_*n*_^+^ clusters during sputtering with Cs^+^) and negative secondary ions (recorded during sputtering with Cs^+^) does not always provide enough results to be able to draw unambiguous conclusions. We should emphasize that we also used Ar^+^ ions for etching, but O_2_^+^ and Cs^+^ ions appeared to be the better choice. For the FeAgNi and CrTiAl samples, etching with Ar^+^ is not suitable because of the problems with the low intensity of the positive secondary ions. Moreover, the positions of their maxima with respect to the metal and metal oxide layers are not correct, similar to the O_2_^+^ etching. The intensities of the negative secondary ions are even lower and, in some cases, indistinguishable from the noise. In the case of Ar^+^ etching of the TiSi sample, both elements have their maxima in the same layers, so it is impossible to distinguish them.

To improve our depth-profiling results, we decided to proceed with gas flooding. Gas flooding with oxygen has often been applied during SIMS analyses to increase the ionization probability of the particles emitted from the surface into positive secondary ions.^76,77^ Besides O_2_, we also introduced gases such as CO, C_2_H_2_, and H_2_ into the analysis chamber. The use of O_2_ gas for flooding in SIMS analyses is a common practice, whereas the application of CO, C_2_H_2_, and H_2_ gases is a novelty of this work, to the best of our knowledge. Our main goals were to minimize the sample-induced matrix effect, increase the secondary-ion yield, and unambiguously resolve the structure of our multilayer samples while recording only one depth profile. Since we wanted to distinguish metals from their oxide layers, etching with O_2_^+^ ions was not acceptable because the metal layer oxidizes during the etching process. O_2_^+^ in combination with gas flooding also introduces two other problems. First, O_2_^+^ can react with many gases, in our case with all those we used (CO, C_2_H_2_, and H_2_). Second, sputtering with O_2_^+^ increases the pressure in the analysis chamber by an order of magnitude, reducing the pressure range in which the flooding gas can be tested.

Nonetheless, we performed the depth profiling of our samples with a Cs^+^ sputtering beam in the presence of O_2_. For the FeAgNi and CrTiAl samples, the O_2_ flooding provides no advantage over Cs^+^ or O_2_^+^ sputtering in a vacuum. For the TiSi sample, slightly larger and more important differences were observed. We chose to analyze the negative secondary ions (Figure S4) since Cs^+^ sputtering enhances the formation of negative ions more than O_2_ flooding enhances the formation of positive ions. Furthermore, as seen in Figure S4, we mainly focused on the oxide species, which ionize better in the negative polarity. Figure S4a shows us that the multilayer structure of the Ti and Si thin layers is resolved, but not ideally since multimaxima structures appear. An important improvement can be seen when analyzing the Ti–Si alloy layers. Namely, the difference between layers with different Ti/Si atomic ratios is now clear. The intensity of the TiO^–^ and TiO_2_^–^ signals decreases as the relative concentration of the Ti decreases and the intensity of the Si^–^ and SiO_2_^–^ signals increases as the relative concentration of the Si increases (Figure S4b). From Figure S4 we can also observe another important feature of O_2_ flooding. This is a significant decrease in the sputtering rate. When other analyzed samples are also included in the consideration, this decrease appears to be between 70 and 300% with respect to measurements without gas flooding.

The main idea of the O_2_ flooding, which has often been applied by various research groups, is to increase the ionization yield. We have extended such studies to the use of other gases such as CO, C_2_H_2_, and H_2_. Inert gases are not suitable for such purposes, so we chose reactive ones. CO is reactive because of the partial negative charge on the C atom and the partial positive charge on the O atom, C_2_H_2_ because of its two π bonds, and H_2_ because of the weak σ bond. We also considered that the species formed should ionize well into the anions because etching with Cs^+^ ions increases the yield of negative secondary ions. When choosing the reactive gas, we also need to make sure that it is not too reactive (e.g., F_2_) to avoid damaging the components of the spectrometer. Besides the increased ionization, our main goal was to exploit the matrix effect in our favor by applying a specific atmosphere, which is the universal matrix independent of the sample to be analyzed. Namely, when the samples are sputtered and the emitted particles are ionized in a vacuum, the only matrix comes from the sample itself and is, therefore, sample specific. However, when we introduce the reactive gas into the analysis chamber, we create an artificial gaseous matrix that is under our control. Such a matrix affects different samples in the same way.

### CO Flooding

**Figure 6:**
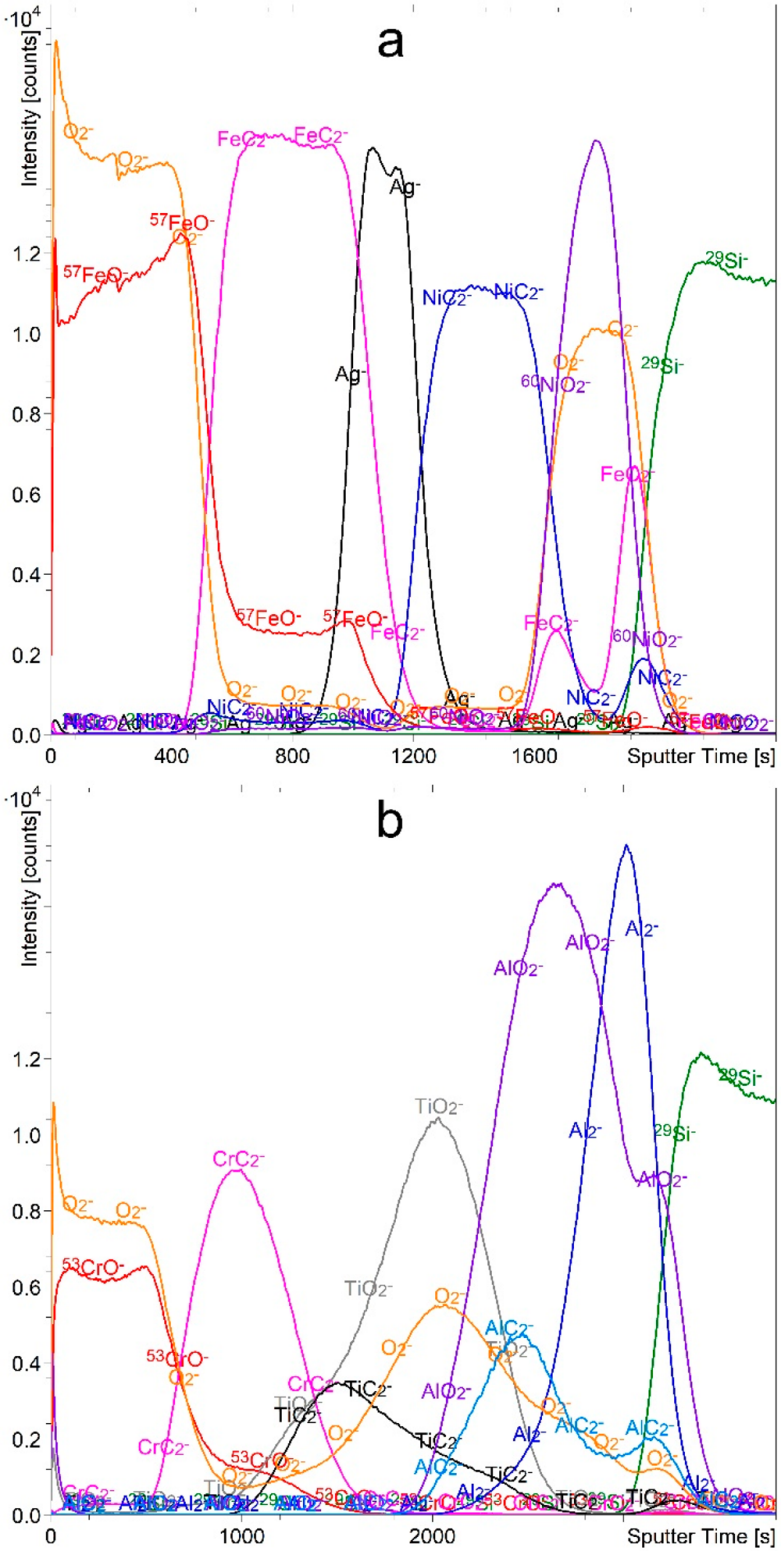
Depth profiles of FeAgNi (a) and CrTiAl (b) samples recorded using a 1 keV Cs^+^ sputtering beam and an atmosphere of 2 × 10^–7^ mbar CO. The intensity-multiplication factors are 0.5 for O_2_^–^, 2.0 for ^57^FeO^–^, 0.6 for ^60^NiO_2_^–^, 0.7 for AlO_2_^–^, 3.0 for AlC_2_^–^, and 0.5 for Al_2_^–^. The reason for the two additional FeC_2_^–^ maxima in the profile of the FeAgNi sample is that Ti contamination on the Si surface as TiO_2_^–^ presents an isobaric interference for the FeC_2_^–^ signal.

We performed depth profiling of the FeAgNi and CrTiAl samples using the Cs^+^ sputter beam in an atmosphere of 2 × 10^–7^ mbar CO (Figure 6). The analysis of the depth profiles of the MC_2_^–^ and MO_n_^–^ signals showed improved depth profiles with moderate depth resolution. In the case of the FeAgNi sample (Figure 6a), all the layers are resolved and the multilayer structure of the sample can be derived from this profile alone, with only minor uncertainties. It should be noted that only weak AgC_*n*_^–^ signals could be detected due to the inertness of the Ag. The depth profile of the CrTiAl sample (Figure 6b) was not successful. The Ti and TiO_2_ layers are difficult to distinguish because there is no sharp interface between them. The AlC_2_^–^ signal has its maximum in the Al_2_O_3_ layer, so the Al and Al_2_O_3_ layers cannot be correctly identified. The AlC^–^ signal shows the same pattern as AlC_2_^–^. As a reactive gas, CO is a suitable choice, but its main drawback is the fact that it consists of C as well as O atoms. The related problems can be seen in Figure 6, where we can see that the signals of the O_2_^–^ and MO^–^ species do not drop to zero in the layers of pure metal. This could mean that the metal layer is partially oxidized, which is not the case.

The results in the case of depth profiling for the TiSi sample with Cs^+^ ions in the CO atmosphere were slightly worse than those obtained in the O_2_ atmosphere. As shown in Figure S5a, the TiO^–^ peak has two local maxima located at the Si/Ti and Ti/Si interfaces. This indicates the presence of an extensive matrix effect at the interfaces, which has a negative effect on the profile structure. The SiO_2_^–^ signal similarly shows an unclear multimaxima structure. The TiC_2_^–^ signal is very weak and also does not clearly resolve the multilayer structure. The Ti–Si alloy layers in the TiSi sample offer a slightly better result. As the relative concentration of silicon increases, the intensity of the SiC^–^ and SiO_2_^–^ signals increases as well. As the relative concentration of titanium decreases, the intensity of the TiO^–^ signal also decreases, while the intensity of the TiC_2_^–^ signal remains more or less constant (Figure S5b). Nevertheless, we should emphasize that the CO flooding reduces the sputtering rate by 20–90% (depending on the sample), which is much less than in the case of the O_2_ atmosphere.

### C_2_H_2_ Flooding

The presence of acetylene in the analysis chamber results in depth profiles similar to those recorded when using CO. Positive results are obtained as the intensity of the MO_*n*_^–^ secondary ions decreases in the layers of pure metal, compared to the CO flooding (Figure S6), due to the absence of oxygen in the C_2_H_2_ molecule. An exception is seen for Al in the CrTiAl sample. The layer of “pure” Al is a mixture of Al and Al_2_O_3_ because aluminum is partially oxidized during the sample preparation due to its reactivity (Figure S6b). Oxide layers can therefore be identified from the O^–^, OH^–^, C_*n*_O_*m*_^–^, and C_*n*_O_*m*_H^–^ species. However, in contrast to the application of CO, no significant reduction of the MC_2_^–^ signal’s intensities is observed in the oxide layers in the case of C_2_H_2_ flooding. Additionally, the AlC_2_^–^ signal again shows its maximum in the Al_2_O_3_ layer (Figure S6b). Even worse results are obtained when observing the MC^–^, MH_*n*_^–^, and MC_*n*_H^–^ signals, since their intensities are more or less constant through both the pure metal and its oxide layers. Al is again an exception, with the maxima in the Al_2_O_3_ layer. From the comparison of the depth profiles in Figures 6 and S6, it appears that, at least for the FeAgNi and CrTiAl samples, the CO atmosphere is a better choice than the C_2_H_2_ atmosphere, since the layers are much more clearly resolved. An improvement in the case of the C_2_H_2_ atmosphere can only be seen when discriminating between the Ti and TiO_2_ layers.

There is also not much difference or improvement for the TiSi sample compared to the CO flooding. As can be seen in Figure S7a, both the SiC^–^ and SiH^–^ signals clearly indicate the positions of the Si layers. The depth profile of the TiC_2_^–^ signal has its maxima in the Ti layers, but they are not as pronounced as in the case of the Si species. Moreover, additional smaller local maxima from the TiC_2_^–^ signal can be observed in the Si layers. The intensity of both the SiC^–^ and SiH^–^ signals increases with an increase in the relative concentration of the Si in the Ti–Si alloy layers (Figure S7b). We can also observe that the intensity of the TiC_2_^–^ signal decreases as the relative Ti concentration decreases. Therefore, a C_2_H_2_ atmosphere is more suitable for the analysis of Ti–Si alloys than CO. Nevertheless, we also observe a larger decrease in the sputtering rate compared to the CO flooding (between 30 and 150%, depending on the sample). Based on these measurements, we can conclude that neither O_2_, CO, or C_2_H_2_ is an ideal choice that would provide excellent layer resolution with a clearly resolved multilayer structure for our samples.

### H_2_ Flooding

**Figure 7:**
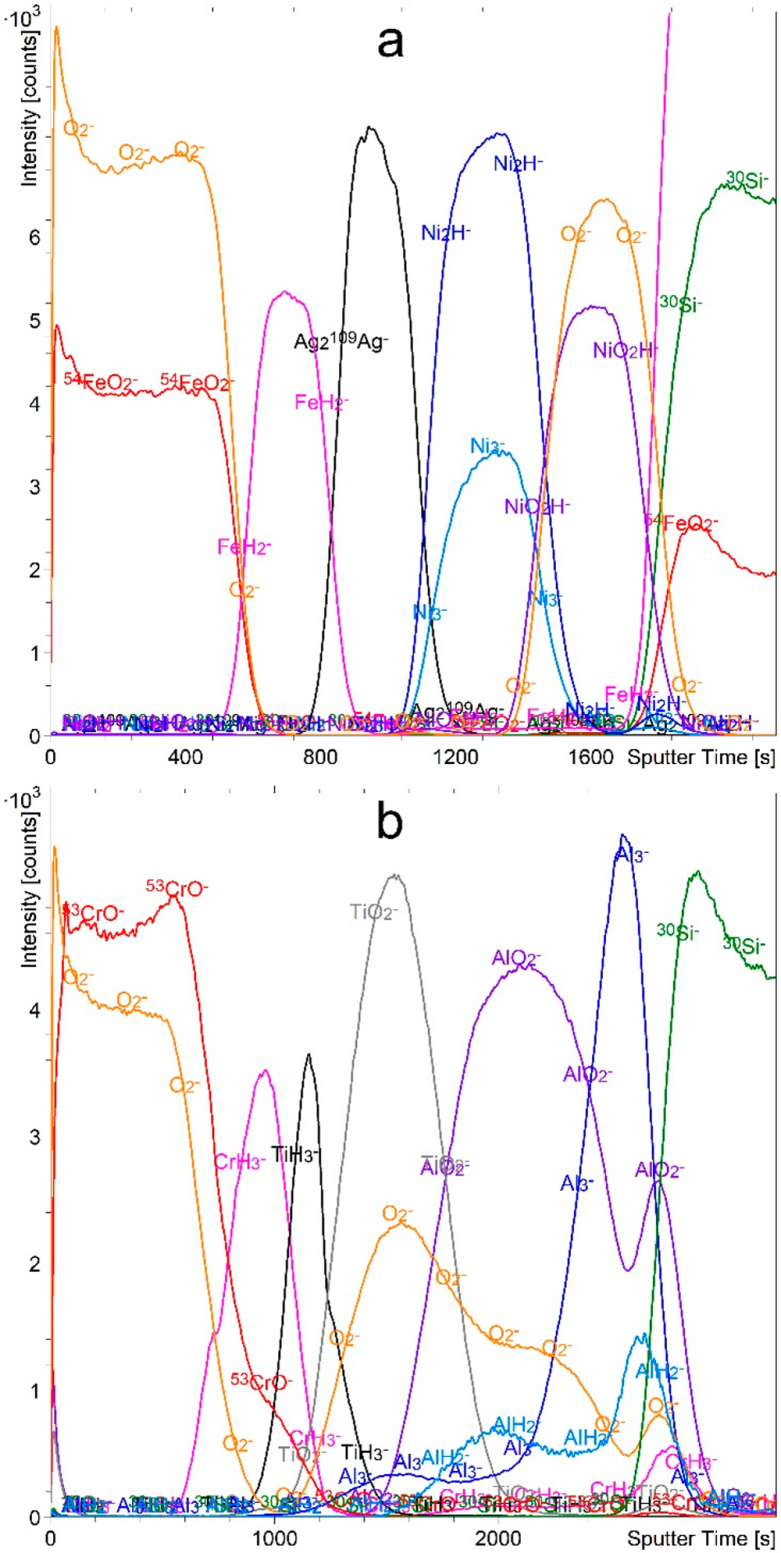
Depth profiles of FeAgNi (a) and CrTiAl (b) samples recorded using a 1 keV Cs^+^ sputtering beam and an atmosphere of 7 × 10^–7^ mbar H_2_. The intensity-multiplication factors are 0.3 for O_2_^–^, 0.4 for Ni_2_H^–^, 0.4 for NiO_2_H^–^, 0.7 for ^53^CrO^–^, 0.6 for TiO_2_^–^, 0.2 for AlO_2_^–^, 5.0 for AlH_2_^–^, 0.5 for Al_3_^–^, and 0.7 for ^30^Si^–^. The reason for the intensity increase of the FeH_2_^–^ and ^54^FeO_2_^–^ signals in the Si substrate for the profile of the FeAgNi sample is the overlapping of these signals with the Si_n_^–^ signals of different silicon isotopes.

Finally, we performed depth profiling with H_2_ flooding at a pressure of 7 × 10^–7^ mbar. By using hydrogen, we eliminate the presence of oxygen and simplify the analysis by introducing only one new element. This also means that we can perform a reliable analysis of all the elements except hydrogen. Indeed, the results for both the FeAgNi and CrTiAl samples are very promising. As shown in Figure 7, when the H_2_ gas was introduced into the analysis chamber, we were able to record depth profiles that correctly and unambiguously represent the exact multilayer structure of our samples. The maxima of all the M_*n*_*H*_*m*_^–^ signals are located in the layers of pure metal, so we can preferentially choose the most intense of them. M_*n*_^–^ metallic cluster signals also have maxima in the metal layers. The metal oxide layer can be identified from either MO_*n*_^–^ or MO_*n*_H^–^ signals. We should also emphasize that the depth resolution is improved compared to all the other atmospheres. As mentioned before, the presence of the AlO_2_^–^ signal in the Al layer (Figure 7b) is due to the unwanted oxidation that occurred during the sample preparation. It should also be mentioned that most metals form very intense M_*n*_H_*m*_^–^ signals. Exceptions are Ag, which is inert and does not form clusters with any of the gases used, and Al, whose AlH_*n*_^–^ signals are much weaker than those of the other elements. They are also not as representative of the exact structure as the Al_*n*_^–^ signals.

While flooded with H_2_, very good results were also obtained for the TiSi sample. Figure S8a shows the depth profile of the alternating Ti and Si thin layers. The TiH^–^ and Si_3_^–^ signals are completely separate and resolve all the thin layers as the intensity of both the TiH^–^ in the Si layers and the Si_3_^–^ in the Ti layers decreases close to zero—like during the Cs^+^ sputtering in a vacuum (Figure 4a), where the reason is dissolved hydrogen in the Ti layers. However, the SiH_*n*_^–^ signals (SiH^–^ shown in Figure S8) being the most intense in the Ti layers and having double maxima at both interfaces are problematic due to the matrix effect. The same can be observed, however, for the SiH_*n*_^–^ signals during the Cs^+^ sputtering without gas flooding. Such results can again be explained by the good solubility of hydrogen in titanium.^80,81^ Namely, as seen in Figure S8, the H^–^ signal also has its maxima in the Ti layers, indicating an elevated concentration. Observations of dissolved hydrogen in the Ti layers even in the TiSi samples exposed only to the ambient conditions (Figure 4a) proves this hypothesis even further. Since ion sputtering causes atom mixing, some Si atoms enter the Ti layers, where they can form SiH_*n*_^–^ cluster ions with the hydrogen dissolved in titanium. Furthermore, the first SiH_*n*_^–^ maximum is at the interface between the first Ti and the first Si layer, as no silicon could enter the first Ti layer, which is directly at the sample surface, further supporting the theory of the combined effects of dissolved hydrogen in the Ti layers and sputtering-induced atom mixing. The Si_2_H^–^ signal, however, has three-maxima-shaped signals with the two maxima at each interface and one in the middle of the Si layer. This phenomenon can be explained in a similar way to the previous one. Namely, for the Si_2_H^–^ ion formation, a higher concentration of silicon is needed, and the maxima therefore appear in the middle of the Si layer, where the concentration of silicon is the highest and, on each interface, where the silicon concentration is still sufficient, while the hydrogen concentration starts to rise abruptly due to its solubility in titanium. Nevertheless, the Si_2_H^–^ intensity also remains above zero in the Ti layers. Similarly, the correct as well as the matrix-influenced results for the Ti–Si alloy layers are shown in Figure S8b. As the relative concentration of titanium decreases, the intensity of the TiH^–^ signal decreases as well. As the relative concentration of Si increases, the intensity of the Si_3_^–^ and Si_2_H^–^ signals also increases, while the intensity of the SiH^–^ signal decreases. This happens because of the elevated concentration of hydrogen in the layer with a higher concentration of titanium, also causing the intensity rise of the SiH^–^ signal.

**Figure 8:**
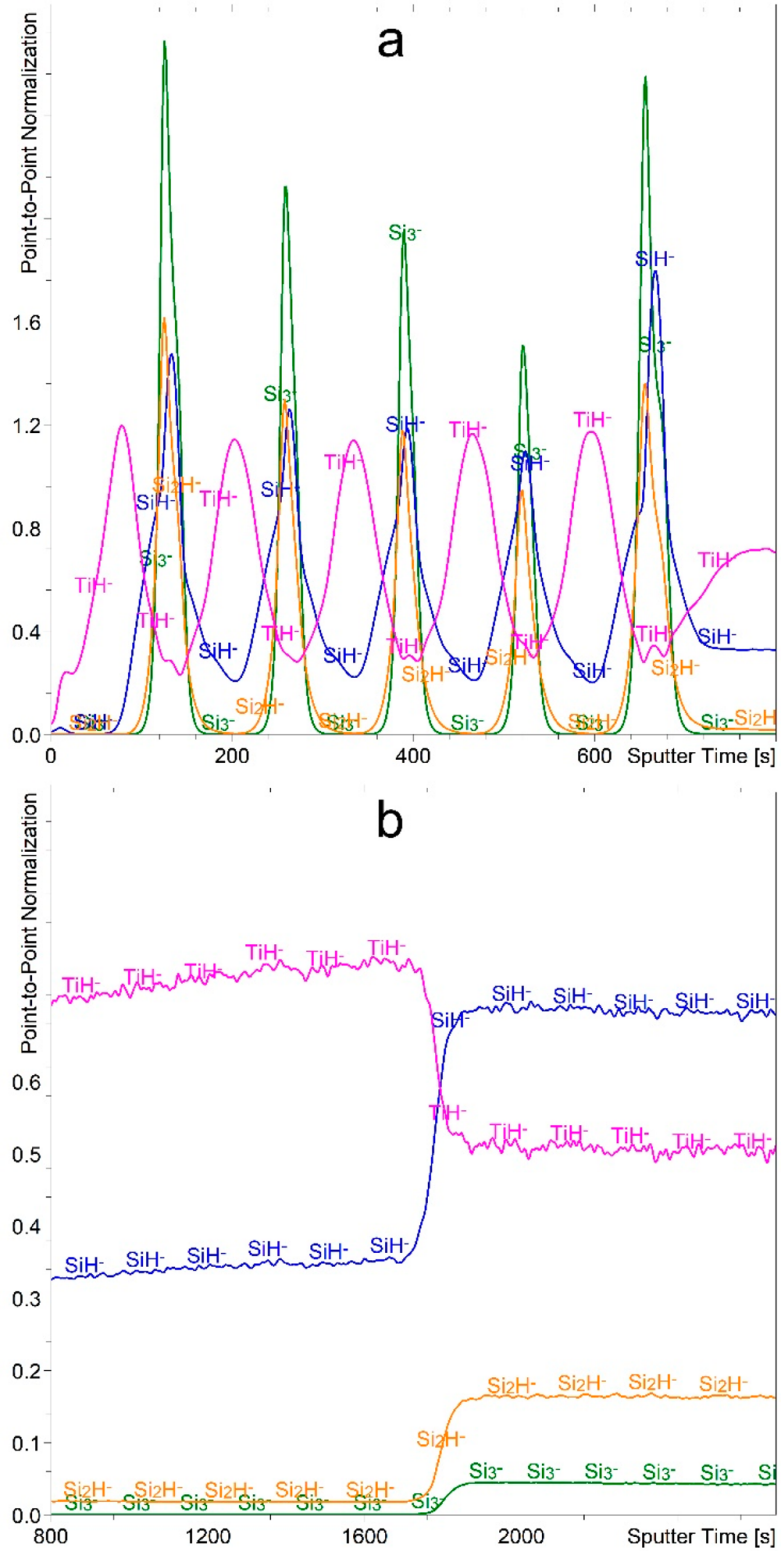
Depth profile of TiSi sample with the scale normalized to the intensity of the H^–^ signal recorded using a 1 keV Cs^+^ sputtering beam and an atmosphere of 7 × 10^–7^ mbar H_2_. The depth profile (a) presents the first 800 s of etching time, while the profile (b) presents the etching time interval between 800 and 2650 s. The intensity-multiplication factor for TiH^–^ is 15.0.

As already mentioned, the matrix effect strongly affects the intensity of the H^–^ ions as well. Therefore, in the next step we considered the normalization of the SIMS signals to the H^–^ signal, as shown in Figure 8. After such a normalization, we obtain an improved profile for the alternating Ti and Si multilayer structure with correctly positioned maxima for all the signals (TiH^–^, Si_3_^–^, SiH^–^, and Si_2_H^–^). The only drawback of this approach is that after such a normalization the intensity of the TiH^–^ signal in the Si layers and the SiH^–^ signal in the Ti layers does not drop to zero. A similar improvement is seen at the interface between the Ti–Si alloy layers, as shown in Figure 8b. As the relative Ti concentration decreases, the intensity of the TiH^–^ signal decreases as well. However, as the relative Si concentration increases, the intensity of all the Si-related signals (Si_3_^–^, Si_2_H^–^, and SiH^–^) also increases.

Therefore, we can conclude that by introducing the H_2_ gas into the analysis chamber in the pressure range of 10^–7^ mbar the multilayer structure of all our samples can be clearly and unambiguously resolved. Moreover, we achieved this while analyzing only a depth profile of negative secondary ions recorded during etching with Cs^+^ ions. We must also emphasize that flooding with H_2_ gas does not significantly affect the sputtering rate for any of our samples, suggesting yet another advantage of hydrogen flooding.

We also tried to use Ar^+^ ions for etching in a H_2_ atmosphere, but the results were again significantly worse than those obtained by sputtering with Cs^+^ ions. Namely, the intensity of the negative secondary ions is too low, similar to that obtained for Ar^+^ sputtering without gas flooding. The intensity of the positive secondary ions is slightly higher, but still around 2 orders of magnitude lower than the intensity of the negative secondary ions recorded while sputtering with Cs^+^. Also, the multilayer structure is less pronounced and the differentiation between layers less clear than in the depth profiles recorded with the Cs^+^ sputter ion beam.

### Cluster Secondary-Ion Formation

A recent study has shown that the adsorption of gaseous species on a sample surface is very fast and happens during the ion-etching process as well.^86^ We believe, therefore, that the O_2_, CO, C_2_H_2_, and H_2_ become adsorbed on the freshly exposed sample surface between the Cs^+^ sputtering cycles but do not penetrate extensively into the bulk. The exception is the TiSi sample in a H_2_ atmosphere, as in this case, as already presented (Figure 4), hydrogen becomes dissolved in all the thin Ti layers, even when the sample is only exposed to ambient conditions. Such claims are supported by three observations. Namely, if we lower the gas pressure in the analysis chamber, the intensity of the cluster secondary-ion species such as hydrides or carbides will immediately decrease in proportion to the pressure reduction. If the gases were to penetrate deeper into the bulk, as seen for hydrogen and the TiSi sample, the decrease would not be so sudden. Furthermore, a significant decrease in the sputter rate during O_2_ flooding can be explained via the O_2_ adsorption and the formation of thin oxide layers. As seen in Figure 3, the sputter rate of the metal is greater than the sputter rate of its oxide, since all the layers are of similar thickness. The oxide layers formed on the surfaces of the metals in the O_2_ atmosphere have a lower sputter rate, and consequently, the analysis time is prolonged, indicating a reduced sputter rate. The greatest prolongation of the analysis time was seen for the case of the TiSi sample, which initially has no oxide layers. Since the sputter rate changes for the metal layers, but not for the metal oxide layers, this observation confirms our explanation. Figures 6 and S6 indicate that metal carbides have a lower sputter rate compared to the pure metal as well, explaining the moderate reduction in sputter rate during CO and C_2_H_2_ flooding. Nevertheless, Figure 7 indicates that metal hydrides do not differ significantly from the metals in terms of the sputter rate, so no significant change in the sputter rate for any of our samples is expected while flooding with the H_2_ gas, matching our experimental results. Observations made during H_2_ flooding are supported by the well-known embrittlement of metals resulting from hydrogen adsorption because the structure of the hydride is less stable.^81,87,88^

Finally, we also noticed that the intensity of many secondary ions, which are not formed as a consequence of the recombination with the gas molecules (Fe^–^, Ni^–^, Cr^–^, Al^–^, Al_2_^–^, Al_3_^–^, Si^–^, Si_2_^–^, Si_3_^–^), does not change significantly in comparison with the Cs^+^ sputtering in vacuum, regardless of the gas used. Therefore, the processes responsible for the cluster secondary-ion formation observed in our analyses most probably take place exclusively on the surface or just above the surface, in both cases specifically during ion sputtering. Major chemical changes caused by the absorption would probably also cause the changes in the intensity of the mentioned secondary ions, which is not the case. The intensity change of the secondary-ion species connected with the hydrogen dissolved in the TiSi sample provides additional support for such a hypothesis. Our prediction about the cluster-ion origin is further supported by extensive studies concerning the mechanism of their formation, proved by both the computer simulations and the experimental findings. As we were bombarding metallic and ionic (oxide) surfaces with monatomic ions, a collision cascade is the appropriate approximation of the processes involved.^89^ Cluster ions formed during a collision cascade are mainly formed from the atoms that were initially first or second neighbors at the surface.^89,90^ During ion sputtering, they move as a bound cluster in the selvedge region of the surface, followed by desorption into the gas phase.^91,92^ Another, yet similar, explanation is that the cluster-ion formation happens in the near-surface region,^90,93,94^ probably as the recombination of a neutral atom and a sputtered ion.^94^ Recombination above the sample surface is also the supposed reason for the matrix effect’s reduction.^94^ Both of described mechanisms correspond to our hypothesis that cluster secondary ions composed of metals and flooding gases are formed on the topmost surface layers or just above the surface after the gas molecule’s adsorption, always during the ion-sputtering process.

Last but not least, the reasons behind the greatest improvement in the depth profiles achieved during H_2_ flooding have not been proven yet. Nevertheless, we believe that the specificity of the chemical reactions of the different gases is at play, at least to some degree. Figures S6 and S7 show a significant intensity decrease in the metal oxide signals in the metal layers during the C_2_H_2_ and H_2_ flooding. The same can be seen for the Cs^+^ sputtering without gas flooding (Figure 3). Such observations are expected since no oxygen source is present. In contrast, during the CO flooding (Figure 6), the metal oxide signal’s intensity stays significant in the metal layers as well due to the CO being a source of both carbon and oxygen. Even more important is the observation of the intensity of the signals in the metal oxide layers. Namely, the intensity of the metal hydride signals decreases to near-zero in the metal oxide layers (Figure 7), while this is not the case for the metal-carbide signals (Figures 6 and S6). The most probable explanation for this is a different mechanism for the reactions with hydrogen and carbon. It appears that hydrogen preferentially forms hydroxides with metal oxides, while carbon rather substitutes for oxygen in the metal oxides, forming metal carbides in a similar manner as in the layers of pure metals. The greatest difference between the intensity of the secondary ions in metal and metal oxide layers in the presence of H_2_ among all the atmospheres tested is therefore a consequence of the different reaction mechanisms. Nevertheless, these processes cannot fully explain the improved interface resolution observed during H_2_ flooding.

## Conclusion

Our study shows that introducing different gases into the analysis chamber during SIMS depth profiling can lead to very different depth profiles with respect to the type of gas used. As a result, new information has been obtained. We have found that when using O_2_, CO, or C_2_H_2_ there is an improvement with respect to a vacuum, but only in some specific cases. Therefore, at least two separate depth profiles are needed to explain the results of the analysis correctly and with sufficient confidence. However, since the introduction of these gases also reduces the sputtering rate, the time required for such an analysis is even greater than when two profiles are recorded with two different types of etching ions. In contrast, the introduction of H_2_ does not reduce the sputtering rate and shows improved results for all the samples we tested. By only recording the depth profile of the negative secondary ions while using the Cs^+^ sputter ion beam and H_2_ flooding, we were able to determine the compositional depth of FeAgNi, CrTiAl and TiSi samples while clearly distinguishing between successive layers.

SIMS dual-beam depth profiling in a hydrogen atmosphere in the range of 10^–7^ mbar thus appears to be a successful approach for the analysis of metal, metal oxide, and alloy multilayers. In this way, the interfaces between different metals, metals and their oxides, and different alloys can be determined with a satisfactory depth resolution. It also offers the potential for the analysis of other elements with sufficiently intense secondary anions. Since only one new, nonharmful element is introduced, the analysis does not become complicated. With this approach, sufficient information for the sample’s structure determination can be obtained with only a single depth profile. Further studies are necessary to analyze the influence of the hydrogen atmosphere on the chemistry and topography of the crater formed during ion etching and also in relation to the depth resolution.

## Supporting Information Available

TEM images, depth profiles (Figures S1–S8) (PDF)
