# Supplementary material for: ToF-SIMS Depth Profiling of Metal, Metal Oxide, and Alloy Multilayers in Atmospheres of H2, C2H2, CO, and O2
Source: J Am Soc Mass Spectrom. 2021 Dec 22;33(1):31–44. doi: 10.1021/jasms.1c00218 (PMC8739835; doi:10.1021/jasms.1c00218)
Supplement: Supplementary file 1 — js1c00218_si_001.pdf [file js1c00218_si_001.pdf]

Supporting information

ToF-SIMS Depth Profiling of Metal, Metal-Oxide and Alloy Multilayers in Atmospheres of H_2, C_2H_2, CO and O_2

Jernej Ekar,[1,2] Peter Panjan,[1] Sandra Drev,[1,3] Janez Kovač[1,*]

[1]Jožef Stefan Institute, Jamova cesta 39, SI-1000 Ljubljana, Slovenia
[2]Jožef Stefan International Postgraduate School, Jamova cesta 39, SI-1000 Ljubljana, Slovenia
[3]Center for electron microscopy and microanalysis, Jamova cesta 39, SI-1000 Ljubljana, Slovenia

Corresponding Author

* Janez Kovač
Jožef Stefan Institute, Jamova cesta 39, SI-1000 Ljubljana, Slovenia
email: janez.kovac@ijs.si

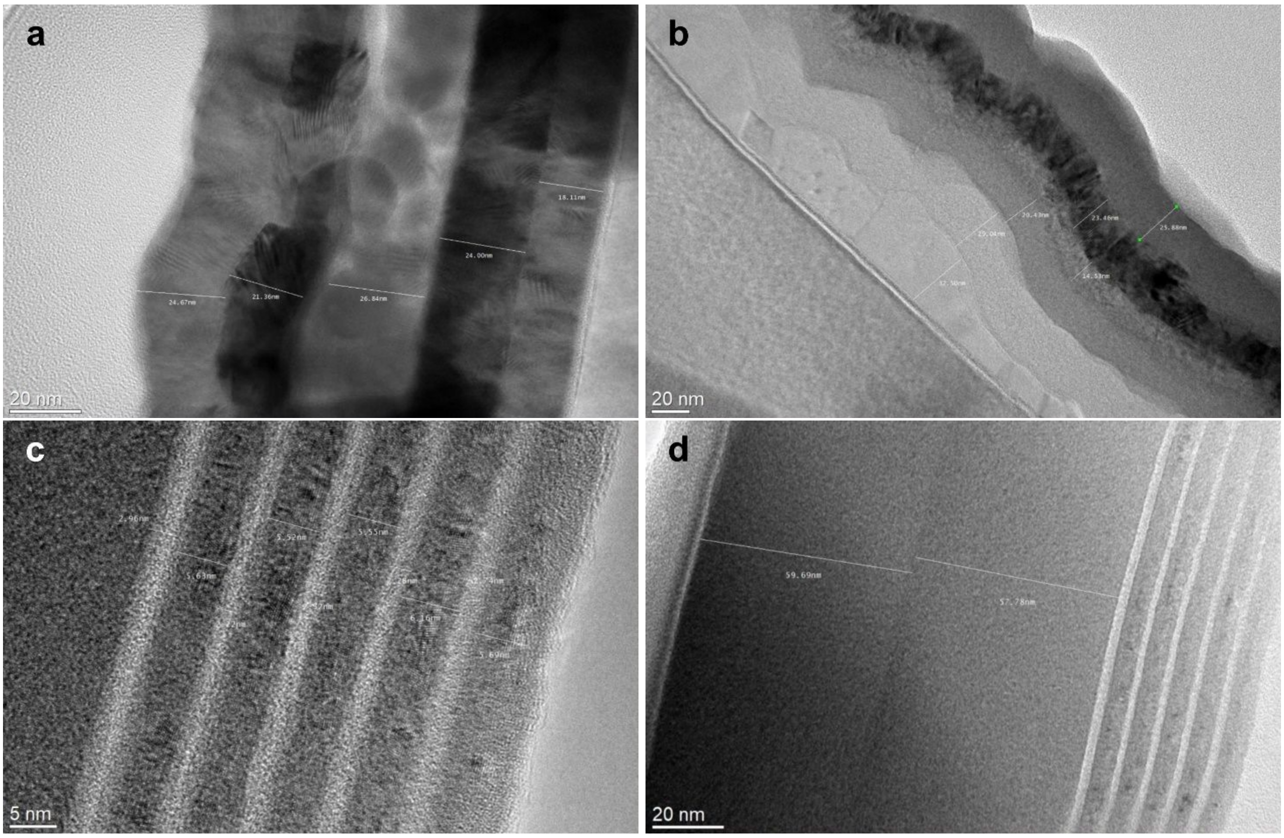

Figure S1. TEM images with the measured thicknesses of the cross sections of the FeAgNi sample (**a**), CrTiAl sample (**b**), thin alternating layers of Ti and Si in the TiSi sample (**c**), and the whole TiSi sample (**d**).

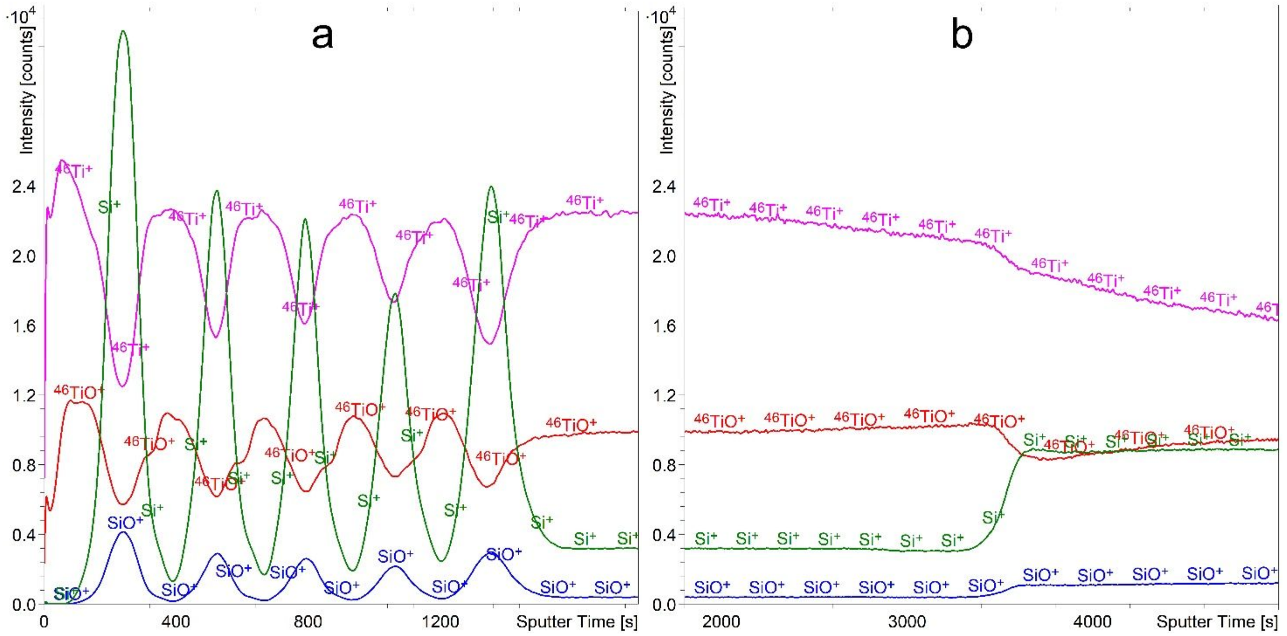

Figure S2. Depth profile of TiSi sample recorded using a 1-keV O_2^+ sputtering beam. The depth profile (**a**) presents the first 1800 s of etching time while the profile (**b**) presents the etching time interval between 1800 and 5000 s. The intensity-multiplication factor for Si^+ is 0.4.

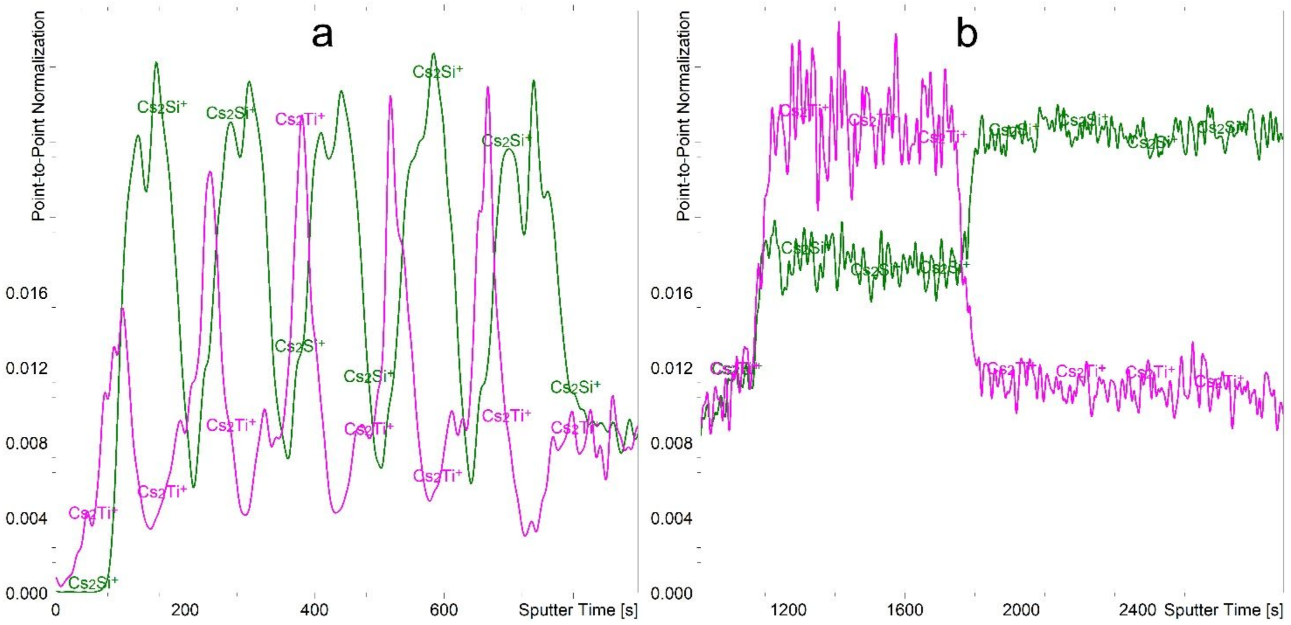

Figure S3. Depth profile of TiSi sample with the scale normalized to the intensity of the Cs_2^+ signal recorded using a 1-keV Cs^+ sputtering beam. The depth profile (**a**) presents the first 900 s of etching time while the profile (**b**) presents the etching time interval between 900 and 2900 s. The intensity-multiplication factor for Cs_2Ti^+ is 4.0.

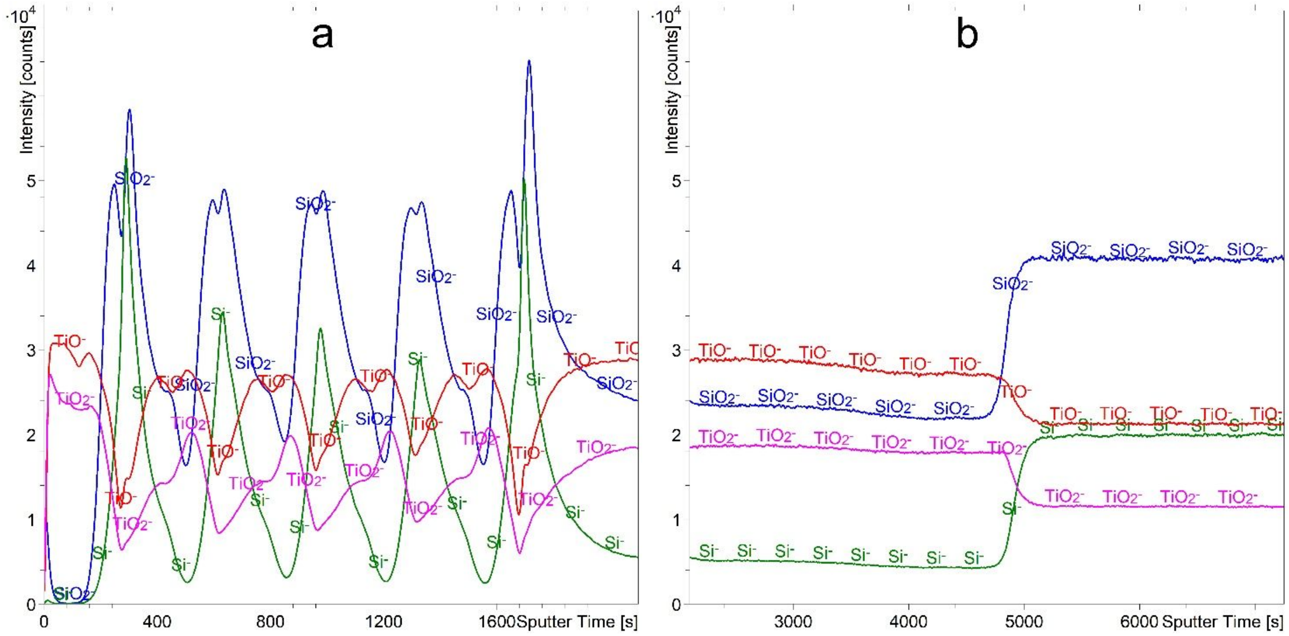

Figure S4. Depth profile of TiSi sample recorded using a 1-keV Cs^+ sputtering beam and an atmosphere of $8{\cdot}10^{-8}$ mbar O_2. The depth profile (**a**) presents the first 2100 s of etching time while the profile (**b**) presents the etching time interval between 2100 and 7250 s.

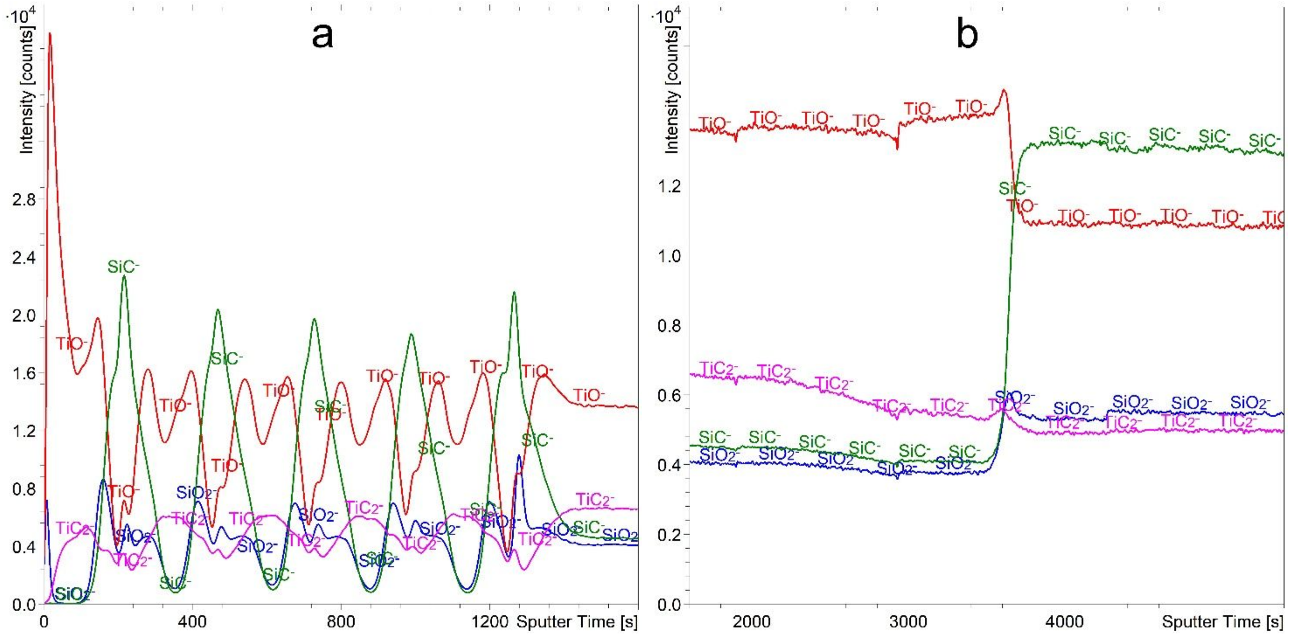

Figure S5. Depth profile of TiSi sample recorded using a 1-keV Cs^+ sputtering beam and an atmosphere of $2{\cdot}10^{-7}$ mbar CO. The depth profile (**a**) presents the first 1600 s of etching time while the profile (**b**) presents the etching time interval between 1600 and 5400 s. The intensity-multiplication factors are 1.5 for TiC_2^- and 0.5 for SiC^-.

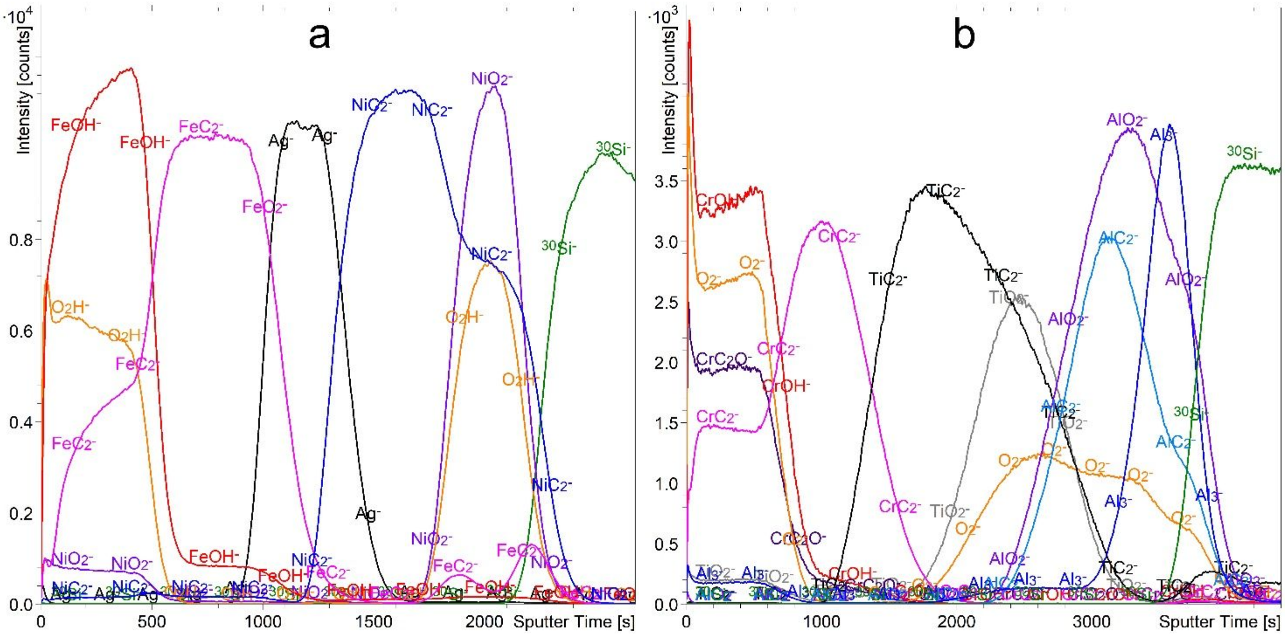

Figure S6. Depth profiles of FeAgNi (**a**) and CrTiAl (**b**) samples recorded using a 1-keV Cs^+ sputtering beam and an atmosphere of $2{\cdot}10^{-7}$ mbar C_2H_2. The intensity-multiplication factors are 0.4 for $FeOH^-$, 0.3 for FeC_2^-, 0.3 for NiC_2^-, 0.4 for O_2^-, 0.2 for CrC_2^-, 0.7 for TiC_2^-, 0.3 for AlO_2^-, 0.2 for AlC_2^-, 0.4 for Al_3^- and 0.4 for $^{30}Si^-$. The reason for the two additional FeC_2^- maxima in the profile of the FeAgNi sample is Ti contamination on the Si surface as TiO_2^- presents an isobaric interference for the FeC_2^- signal.

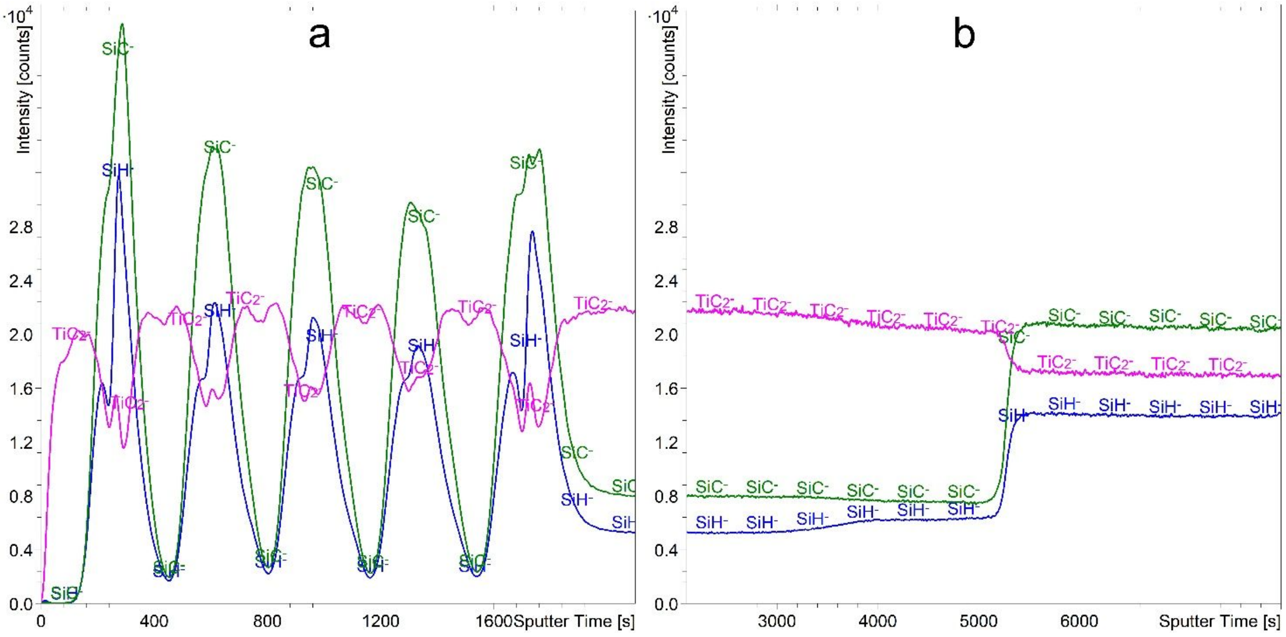

Figure S7. Depth profile of TiSi sample recorded using a 1-keV Cs^+ sputtering beam and an atmosphere of $2{\cdot}10^{-7}$ mbar C_2H_2. The depth profile (**a**) presents the first 2100 s of etching time while the profile (**b**) presents the etching time interval between 2100 and 8000 s. The intensity-multiplication factor for TiC_2^- is 4.0.

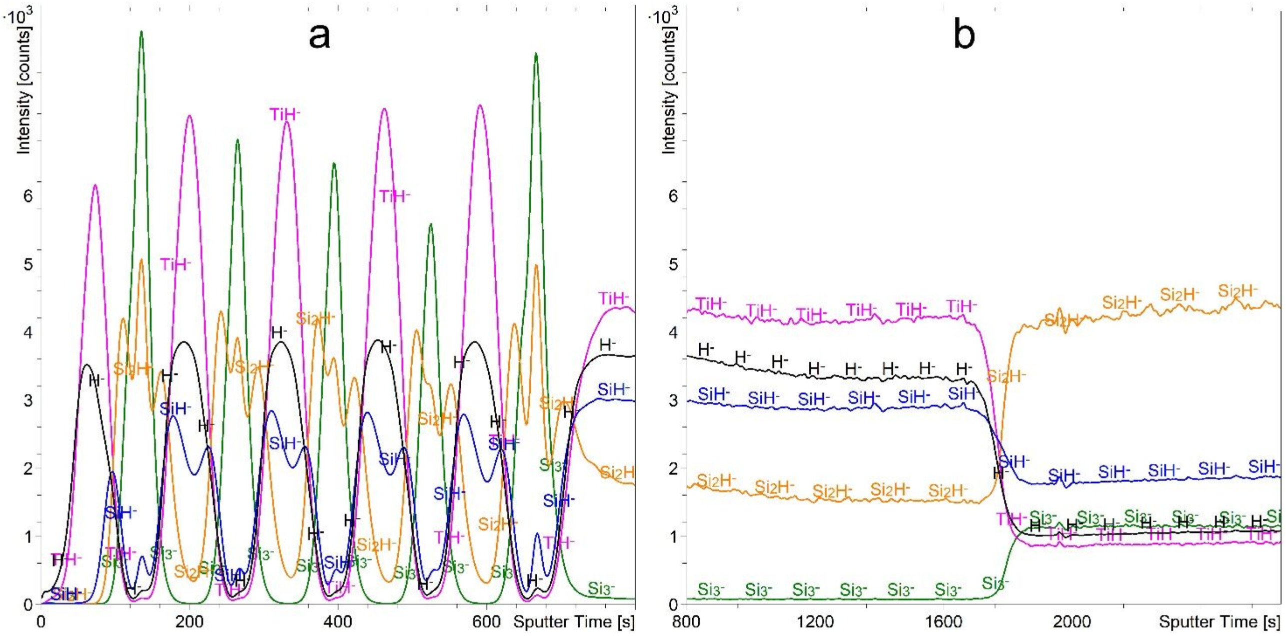

Figure S8. Depth profile of TiSi sample recorded using a 1-keV Cs^+ sputtering beam and an atmosphere of $7{\cdot}10^{-7}$ mbar H_2. The depth profile (**a**) presents the first 800 s of etching time while the profile (**b**) presents the etching time interval between 800 and 2650 s. The intensity-multiplication factors are 0.04 for H^- and 0.1 for SiH^-.
